# Unusual Base Pair between Two 2-Thiouridines and Its Implication for Nonenzymatic RNA Copying

**DOI:** 10.1021/jacs.3c11158

**Published:** 2024-01-31

**Authors:** Dian Ding, Ziyuan Fang, Seohyun Chris Kim, Derek K. O’Flaherty, Xiwen Jia, Talbot B. Stone, Lijun Zhou, Jack W. Szostak

**Affiliations:** † Department of Chemistry and Chemical Biology, Harvard University, 12 Oxford Street, Cambridge, Massachusetts 02138, United States; ‡ Department of Molecular Biology and Center for Computational and Integrative Biology, Massachusetts General Hospital, 185 Cambridge Street, Boston, Massachusetts 02114, United States; § Howard Hughes Medical Institute, Department of Chemistry, The University of Chicago, Chicago, Illinois 60637, United States; ∥ Department of Genetics, Harvard Medical School, 77 Avenue Louis Pasteur, Boston, Massachusetts 02115, United States; ⊥ Department of Chemistry, College of Engineering and Physical Sciences, University of Guelph, Guelph, Ontario N1G 2W1, Canada; # Department of Biochemistry and Biophysics, Perelman School of Medicine, University of Pennsylvania, Philadelphia, Pennsylvania 19104, United States; ∇ Penn Institute for RNA Innovation, University of Pennsylvania, Philadelphia, Pennsylvania 19104, United States

## Abstract

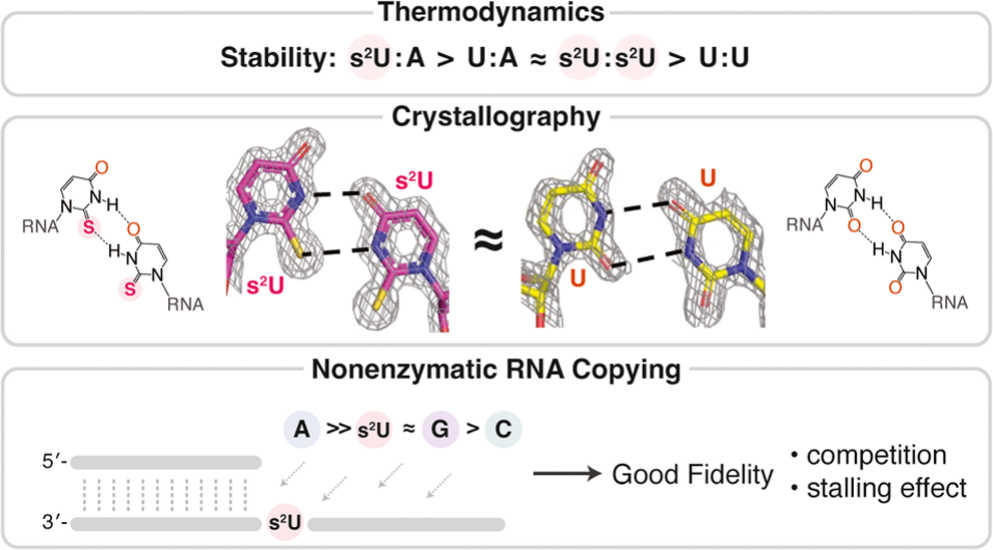

2-Thiouridine (s^2^U) is a nucleobase modification that confers enhanced efficiency and fidelity both on modern tRNA codon translation and on nonenzymatic and ribozyme-catalyzed RNA copying. We have discovered an unusual base pair between two 2-thiouridines that stabilizes an RNA duplex to a degree that is comparable to that of a native A:U base pair. High-resolution crystal structures indicate similar base-pairing geometry and stacking interactions in duplexes containing s^2^U:s^2^U compared to those with U:U pairs. Notably, the C=O···H–N hydrogen bond in the U:U pair is replaced with a C=S···H–N hydrogen bond in the s^2^U:s^2^U base pair. The thermodynamic stability of the s^2^U:s^2^U base pair suggested that this self-pairing might lead to an increased error frequency during nonenzymatic RNA copying. However, competition experiments show that s^2^U:s^2^U base-pairing induces only a low level of misincorporation during nonenzymatic RNA template copying because the correct A:s^2^U base pair outcompetes the slightly weaker s^2^U:s^2^U base pair. In addition, even if an s^2^U is incorrectly incorporated, the addition of the next base is greatly hindered. This strong stalling effect would further increase the effective fidelity of nonenzymatic RNA copying with s^2^U. Our findings suggest that s^2^U may enhance the rate and extent of nonenzymatic copying with only a minimal cost in fidelity.

## Introduction

Post-transcriptional nucleobase modifications of RNAs are common in modern biology, where they confer enhanced functionality on RNAs. Among the many natural RNA modifications, the 2-thiolation of uridine (s^2^U) is particularly interesting, as it is only found in tRNA where its presence is universally conserved across all organisms.^1−3^ Additionally, thiolation in tRNA is preserved even in attempts to generate minimal genomes,^4,5^ possibly because this modification is critical for response to environmental stress.^4−8^ Such widespread conservation suggests a strong evolutionary pressure to maintain the s^2^U modification, which, in principle, could have originated from prebiotic chemistry.

Compared to the structurally flexible uridine, 2-thiouridine adopts a more rigid C3′-endo conformation. This constraint on the conformation of the modified nucleotide also influences the conformation of the adjacent nucleotides; together these effects reinforce A-form geometry in single-stranded RNA.^9^ Additionally, the 2-thiolation of uridine favors Watson–Crick base-pairing with adenosine and disfavors wobble base-pairing with guanosine. As a result, 2-thiouridine can stabilize the tRNA anticodon stem-loop and the codon–anticodon interaction, allowing for improved efficiency and fidelity in codon translation.^10−12^

The enhanced stability and specificity of s^2^U:A base-pairing and the conservation of 2-thiouridine throughout modern biology have prompted researchers to investigate its potential roles in prebiotic chemistry. Our laboratory has previously demonstrated that the nonenzymatic copying of RNA is faster with s^2^U than with U on A-rich templates, and exhibits lower error rates on G-rich templates.^13^ In addition, ribozyme-catalyzed RNA copying with 2-thiolated-U substrates or templates also exhibits an improved rate and fidelity.^14^ Furthermore, a prebiotically plausible synthesis of 2-thiouridine and its derivatives has been demonstrated. Indeed, the 2-thio-pyrimidine nucleosides now appear to be intermediates in the prebiotic synthesis of the canonical nucleotides.^15,16^

Interestingly, further research reveals that the significantly enhanced reactivity of s^2^U in chemical RNA copying is probably not solely due to the stronger s^2^U:A base pair. Indeed, the binding affinity of the s^2^U nucleotide to the RNA template is only modestly improved for nonenzymatic RNA copying (2-fold decrease in *K*_M_ after the s^2^U modification).^17^ This finding suggests that while s^2^U can significantly stabilize RNA duplexes, it may not confer similarly enhanced binding of substrates for nonenzymatic primer extension. Instead, the enhanced reactivity of the thiolated uridine substrate may also be due to the preferred phosphate position conferred by the C3′-endo sugar pucker conformation, which brings it closer to the primer 3′–OH nucleophile.^17^

During our continued studies of s^2^U, we recently discovered that RNA duplexes containing s^2^U:s^2^U mis-pairs are surprisingly stable. Because this unusual s^2^U:s^2^U base pair could strongly influence nonenzymatic RNA template copying, we studied its structure, thermodynamics, and effects on copying chemistry. Here, we report our thermodynamic and crystallographic studies of RNA–RNA duplexes with s^2^U:s^2^U base pairs and provide a rationale for the observed strong interaction. We then evaluated the fidelity of copying of s^2^U-containing templates with s^2^U as the incoming nucleotide substrate. Even though s^2^U is efficiently incorporated opposite s^2^U in the template, we find that in competition experiments, s^2^U is outcompeted by A. Because of the strong stalling effect following an s^2^U:s^2^U mismatch, we conclude that the s^2^U:s^2^U interaction will not greatly reduce the effective fidelity of nonenzymatic template copying.

## Results

### Thermodynamic Analysis of RNA Duplexes Containing s^2^U:s^2^U Base Pairs

We first evaluated the stability of the s^2^U:s^2^U base pair by measuring the melting temperature (*T*_m_) of a 9-bp RNA duplex containing a central U:A, s^2^U:A, U:U, s^2^U:U, or s^2^U:s^2^U base pair, flanked on both sides by four Watson–Crick base pairs (Table 1). *T*_m_ values were measured by variable temperature UV absorbance in 10 mM Tris-HCl at pH 8.0, 1 M NaCl, and 2.5 mM EDTA, at a series of concentrations ranging from 1.25 to 15 μM total RNA. Consistent with our previous report, we found that s^2^U significantly increases the melting temperature of both the A:U base pair and the U:U mis-pair. Strikingly, an s^2^U:s^2^U-containing duplex exhibits a melting temperature almost 14 °C higher than the corresponding U:U-containing duplex. As a result, the s^2^U:s^2^U-containing duplex exhibits a melting temperature comparable to that of the U:A-containing duplex, but still considerably lower than that of the s^2^U:A-containing duplex (Table 1 and Figures S1 and S2).

**Table 1:** Thermodynamic Parameters of RNA Duplex Formation by Thermal Denaturation

			*T*_m_^a^(°C)		Δ*H*^b^(kcal mol^–1^)		Δ*S*^b^(kcal K^–1^ mol^–1^)		Δ*G*_25°C_^c^(kcal mol^–1^)	
group	base pair	duplex sequence	average	SD	average	SD	average	SD	average	SD
1	U:A	5′-CUGA **U** GUAG-3′	46.9	0.2	–78.6	5.4	–0.219	0.017	–13.4	0.4
		3′-GACU **A** CAUC-5′								
2	s^2^U:A	5′-CUGA **s**^**2**^**U** GUAG-3′	52.9	0.1	–80.4	3.2	–0.220	0.010	–14.9	0.3
		3′-GACU **A** CAUC-5′								
3	U:U	5′-CUGA **U** GUAG-3′	31.8	0.2	–66.2	1.1	–0.190	0.004	–9.5	0.1
		3′-GACU **U** CAUC −5′								
4	s^2^U:U	5′-CUGA **s**^**2**^**U** GUAG-3′	39.5	0.1	–74.5	3.1	–0.211	0.010	–11.5	0.2
		3′-GACU **U** CAUC-5′								
5	U:s^2^U	5′-CUGA **U** GUAG-3′	40.3	0.2	–74.0	4.1	–0.209	0.013	–11.7	0.2
		3′-GACU **s**^**2**^**U** CAUC-5′								
6	s^2^U:s^2^U	5′-CUGA **s**^**2**^**U** GUAG-3′	45.4	0.2	–73.4	3.5	–0.203	0.011	–12.7	0.2
		3′-GACU **s**^**2**^**U** CAUC-5′								

a The reported *T*_m_ was calculated from sigmoidal curves of raw thermal UV–vis data at 5 μM total oligonucleotide, 10 mM Tris-HCl 8.0, 1 M NaCl, and 2.5 mM EDTA (Supporting Figure S1).

b Δ*H and* Δ*G* were derived from linear fits of Van’t Hoff plots of *T*_m_^–1^ versus In(*C_T_*/4), where *C_T_* is the total oligonucleotide concentration (Supporting Figure S2).

c Δ*G*_25°C_ was calculated from Δ*H and* Δ*G* according to the equation Δ*G =* Δ*H – T*Δ*S*, where *T* = 298.15 K. Standard errors (N ≥ 7) are reported.

To investigate the origin of the strong stabilization effect induced by thiolation, we evaluated the thermodynamic parameters Δ*H*, Δ*S*, and Δ*G* by fitting the measured melting temperatures at different oligonucleotide concentrations to the Van’t Hoff equation. We found that substituting an A:U base pair with a U:U mis-pair leads to duplex destabilization by almost 4 kcal/mol. This destabilization appears to be the net effect of a very strong enthalpic destabilization (12.4 kcal/mol), which is partially compensated by an entropic gain of 8.6 kcal/mol. Interestingly the free energy penalty of U:U pairing is partially rescued when the uridine on one strand is thiolated and is almost completely rescued when both uridines are substituted with s^2^U.

We suggest that the observed stabilization may result from a reduced desolvation penalty for s^2^U during strand annealing combined with greater strand preorganization prior to annealing. During the RNA hybridization event, each of the two complementary ssRNAs must dissociate from bound water molecules and rearrange sugar configurations into the A-form geometry before forming the double-stranded RNA helix. Given that thioketones are weaker H-bond acceptors than ketones, the energy required to dissociate bound water molecules during hybridization should be reduced.^18^ During the desolvation process, once one water molecule is removed from the hydrogen bond network, cooperative effects may reduce the energetic cost of removing an adjacent water molecule.^19^ As a result, the presence of an internal s^2^U may reduce the enthalpic cost of disrupting the bound water network along the single-strand RNAs. Additionally, s^2^U is known to adopt a C3′-endo conformation and can even cause adjacent nucleotides to adopt a similar configuration.^9^ As a result, s^2^U can help to preorganize single-stranded RNA in the A-form geometry found in duplex RNA. This kind of preorganization can reduce the entropic costs of hybridization.^20^ It is likely that all strands with s^2^U modifications benefit from a reduced desolvation penalty and better preorganization but to different extents depending on the sequence and the pairing base identity.

During oligonucleotide hybridization, it is common to observe entropy-enthalpy compensation, where enthalpically more stable interactions may also decrease entropic freedom, and vice versa.^21,22^ This is a well-known phenomenon in aqueous solutions and has been seen in oligonucleotide hybridization,^22−24^ protein folding,^21,25^ and ligand binding.^26,27^ Depending on the sequence design, an s^2^U:A-containing duplex can either be more enthalpically or more entropically favored compared to their unmodified forms.^28−30^ Our measurements also exhibit this compensation. In Table 1, compared to group 3 (native U:U), thiolation of uridine makes the RNA duplexes more enthalpically favored and entropically disfavored. Despite the entropy-enthalpy compensation, the overall free energy penalties after s^2^U substitution are consistent. We see that both the s^2^U:U and U:s^2^U modified duplexes have similar Δ*G*s (group 4 vs 5*)*, while the s^2^U:s^2^U modification makes the duplex comparable in stability to the duplex with a canonical U:A base pair (group 6 vs 1).

Besides the lower desolvation penalty and greater preorganization that reduce the energetic costs of hybridization, the s^2^U:s^2^U base pair may also benefit from stronger stacking interactions with neighboring bases, which would make the resultant duplex enthalpically more stable. Since thermodynamic data alone cannot determine the strength of these interactions, we turned to crystallographic analysis to better understand the s^2^U:s^2^U base pair.

### Crystal Structure Studies of RNA Duplexes Containing s^2^U:s^2^U Base Pairs

Given that the s^2^U:s^2^U base pair significantly stabilizes an RNA duplex compared to a U:U base pair, we asked whether this unusual base pair also affects the duplex structure in ways that can be observed in high-resolution crystal structures. We initially designed the heptamer duplex (5′-UAGC**s**^**2**^**U**CC-3′, 5′-GG**s**^**2**^**U**GCUA-3′) which has the same sequence as that used in our previous structural study of the s^2^U:U pair.^31^ However, no crystal growth was observed after screening more than 380 buffer conditions. We suspect that the modification of both strands in such a short sequence significantly affected crystal packing.^32^

**Figure 1:**
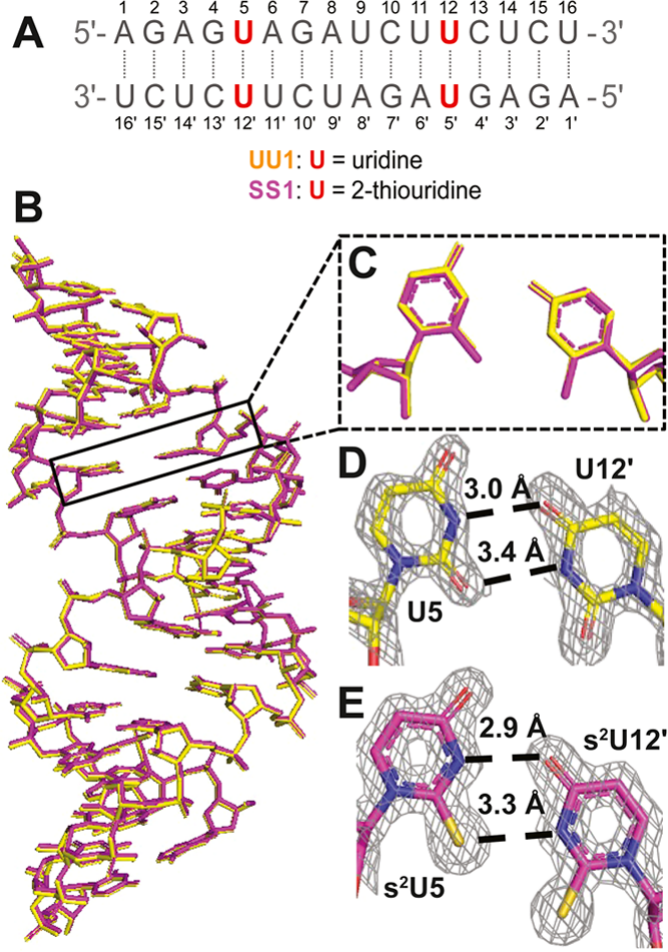
Structures of the UU1 and SS1 duplexes with two separated U:U or s^2^U:s^2^U base pairs. (A) Sequence for UU1 and SS1 (with s^2^U modified at the red position). (B) Superposition of the overall structures for UU1 (yellow) and SS1 (magenta). (C) Comparison of the U:U pair in UU1 (yellow) and the s^2^U:s^2^U pair in SS1 (magenta). (D) U:U base pair in UU1. (E) s^2^U:s^2^U base pair in SS1.The meshes indicate that 2F_o_-F_c_ omit maps contoured at 1.5 σ.

**Figure 2:**
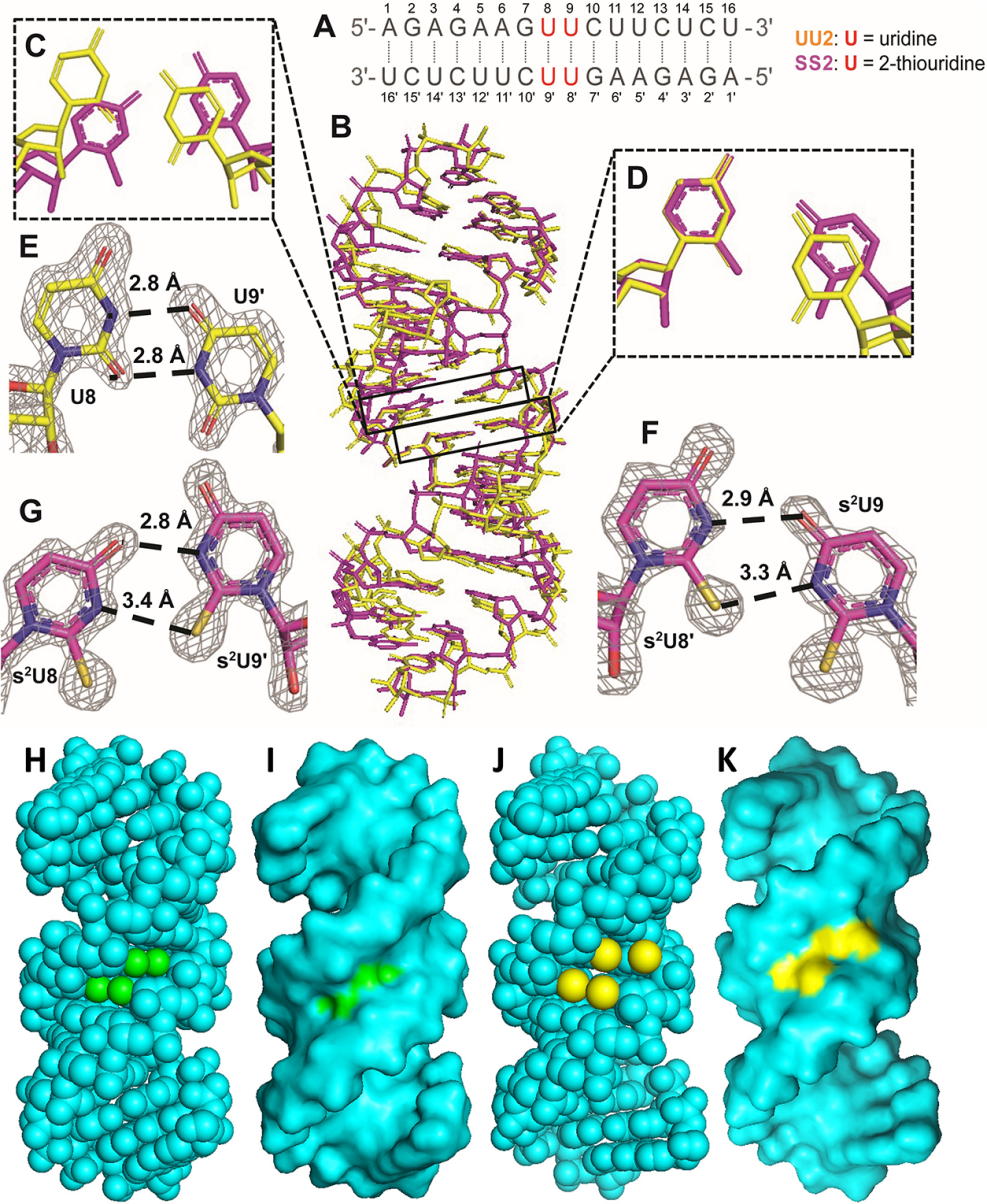
Structures of the UU2 and SS2 duplexes with two adjacent U:U or s^2^U:s^2^U base pairs. (A) Sequence for UU2 and SS2 (with s^2^U modified at the red positions). (B) Superposition of the overall structures for UU2 (yellow) and SS2 (magenta). (C) Comparison of the U8:U9′ pair in UU2 (yellow) and the s^2^U8:s^2^U9′ pair in SS2 (magenta). (D) Comparison of the U9:U8′ pair in UU2 (yellow) and the s^2^U9:s^2^U8′ pair in SS2 (magenta). (E) U:U base pair in UU2. U9:U8′ and U8:U9′ are the same due to symmetry. (F) s^2^U9:s^2^U8′ base pair in SS2. (G) s^2^U8:s^2^U9′ base pair in SS2. Meshes indicate that 2F_o_-F_c_ omit maps contoured at 1.5 σ. (H) Sphere structure for UU2. (I) Surface structure for UU2. O2 atoms of the U:U pairs are displayed in green. (J) Sphere structure for SS2. (K) Surface structure for SS2. S2 atoms of the s^2^U:s^2^U pairs are displayed in yellow.

Therefore, we designed four longer (16-mer) self-complementary RNA sequences based on an original Watson–Crick sequence that led to a high-resolution RNA crystal structure (PDB 3DN4).^32^ We modified this sequence to allow us to study the structural impact of s^2^U:s^2^U base pairs compared to U:U base pairs. The sequence UU1, 5′-AGA G**U**A GAUC U**U**C UCU-3′, can form a duplex with two U/U pairs at the positions of the underlined nucleotides. The two U:U pairs are substituted by two s^2^U:s^2^U pairs in the RNA sequence SS1, 5′-AGA G **s**^**2**^**U**A GAUC U**s**^**2**^**U**C UCU-3′ (Figure 1A). We also designed an RNA sequence, UU2, that forms a duplex with two adjacent U:U pairs, 5′-AGA GAA G**UU**C UUC UCU-3′, as well as its thiolated analog, SS2, 5′-AGA GAA G**s**^**2**^**Us**^**2**^**U**C UUC UCU-3′ (Figure 2A). All four RNAs crystallized within 2–3 days at 20 °C under similar conditions, and all structures were solved at a resolution greater than 1.6 Å. Optimal crystallization conditions are listed in Table S1. Data collection and structure refinement statistics are summarized in Tables S2 and S3. All four structures adopt the same space group (*R*32). The UU1, UU2, and SS1 structures pack similarly, with one strand in each asymmetric unit forming a duplex with another strand in a different asymmetric unit. However, SS2 exhibits a different packing with two strands forming a duplex in one asymmetric unit.

To further understand the impact of the s^2^U:s^2^U base pair on the overall duplex structure and the conformations of the individual nucleotides, we superimposed the respective structures (Figures 1 and 2) and calculated the geometric parameters of all of the base pairs and base pair steps using 3DNA (Tables S4–S11).^33^ The SS1 and native UU1 structures superimpose very well with no significant differences in hydrogen bonding or sugar pucker conformations (Figure 1B). The U:U pair in UU1 forms a shorter hydrogen bond between N3 of U5 and O4 of U12′ compared to the bond between the O2 of U5 and N3 of U12′ (3.0 vs 3.4 Å, Figure 1D). Similarly, the s^2^U5 and s^2^U12′ in SS1 form two hydrogen bonds, one between N3 and O4, and one between S2 and N3 (2.9 Å vs 3.3 Å, Figure 1E). The length of the S2–N3 hydrogen bond in s^2^U:s^2^U (3.3 Å) is similar to that in the s^2^U:U pair reported before (3.4 Å).^31^ Surprisingly, the sulfur substitution did not increase the length of the hydrogen bond in this structure despite the larger atomic radius of the sulfur. However, since a thiocarbonyl is a weaker H-bond acceptor, the strength of the H-bond in the C=S···H interaction could be slightly weaker than that in the C=O···H. Besides the similar H-bond lengths, all of the nucleotides are in the C3′-endo conformation in both structures. We found no significant perturbations between the geometric parameters of UU1 and SS1 (Tables S4–S7). We also calculated the overlap areas of the base pair steps in both sequences to explore the base stacking interactions (Figure S3A). Only a 2.68 Å^2^ difference was observed on the total overlap areas of all base pair steps, which indicates that the s^2^U:s^2^U pair has a negligible impact on base stacking in the crystals.

While the SS1 and UU1 RNAs form very similar structures, the crystal packing of SS2, which contains two adjacent s^2^U:s^2^U base pairs, is slightly different from that of native UU2 (Figure 2B). The U:U pairs in UU2 form two short hydrogen bonds between N3 of U8 and O4 of U9′, and between O2 of U8 and N3 of U9′ (2.8 and 2.8 Å, Figure 2E). In SS2, because there are two strands in one asymmetric unit, the two s^2^U:s^2^U base pairs are not identical, with two hydrogen bonds forming between different pairs of atoms. In the pair s^2^U9:s^2^U8′, the two hydrogen bonds are similar to those of the U:U pair in UU2, with one between the N3 of s^2^U8′ and O4 of s^2^U9, and another between S2 of s^2^U8′ and N3 of s^2^U9 (2.9 and 3.3 Å, Figure 2G). However, the two hydrogen bonds in the pair s^2^U8:s^2^U9′ are between the O4 of s^2^U8 and N3 of s^2^U9′ and between N3 of s^2^U8 and S2 of s^2^U9′ (2.8 and 3.4 Å, Figure 2F). Considering the shorter hydrogen bonds in UU2, the modified s^2^U:s^2^U base pairs in SS2 presumably form weaker hydrogen bonds. Additionally, the distances between the two-position atoms (O or S) are longer in SS2 compared to UU2 (Figure 2H,J), which provides more room for each sulfur atom in SS2. At the same time, the sulfur atoms in SS2 have more surface exposed area compared with the corresponding oxygen atoms in UU2, which are buried in the minor groove (Figure 2I,K). This allows more space in the minor groove of SS2 to accommodate the four sulfur atoms that are close to each other.

Interestingly, although both UU2 and SS2 duplexes maintain the A-form geometry with all nucleotides in the C3′-endo conformation, their structural parameters are slightly different (Tables S8–S11). For instance, the opening of the s^2^U:s^2^U pairs (−4.81°/–1.48°) is much smaller than that of the native U:U pairs (12.83°). This is not surprising because a lower opening angle widens the local minor groove to accommodate the four sulfur atoms on the adjacent s^2^U:s^2^U pairs. In addition, the subtle perturbations of the base pair geometries also impact some local base pair step parameters, especially the twists and overlap areas (Figure S4). The twist on the G7–U8/U9′–C10′ step on SS2 is less than that on UU2 (25.67 vs 42.98°, Figure S4A,B) to provide a suitable angle to form the different hydrogen bonds on the s^2^U8:s^2^U9′ pair. On the other hand, the twist on the U8–U9/U8′–U9′ step on UU2 is smaller than that on SS2 (20.63 vs 37.00°, Figure S4C,D) to allow for the formation of the similarly packed adjacent UU pair. As a result, the overlap area of G7-U8/U9′-C10′ is much smaller in SS2 than that in UU2 (3.86 vs 11.04 Å^2^, Figure S4A,B), indicating a weaker base stacking interaction. These observations suggest that at least in our crystal structures both the hydrogen bonding and base stacking interactions in SS2 are weaker than those in UU2. These changes may be side effects of the duplex structure reorganization required to accommodate the four adjacent sulfur atoms in the minor groove of SS2.

Based on our thermodynamic measurements and our crystallographic observations, we speculate that most of the stabilizing effect of the s^2^U:s^2^U base pair comes from the preorganization of single-stranded RNA before hybridization and the reduced desolvation penalty of s^2^U upon hybridization. Through strand preorganization and reduced desolvation penalty, each s^2^U-containing ssRNA is likely more thermodynamically predisposed for hybridization. Since a duplex with s^2^U:s^2^U base pairs contains s^2^U modifications on each strand, both ssRNAs contribute to the significantly lower Δ*G* of hybridization. Once the duplex is formed, the s^2^U:s^2^U base pair likely has similar or slightly weaker H-bonding and base stacking than the native U/U pair, as suggested by the crystal structures.

### Nonenzymatic Primer Extension with s^2^U in either the Substrate or the Template

**Figure 3:**
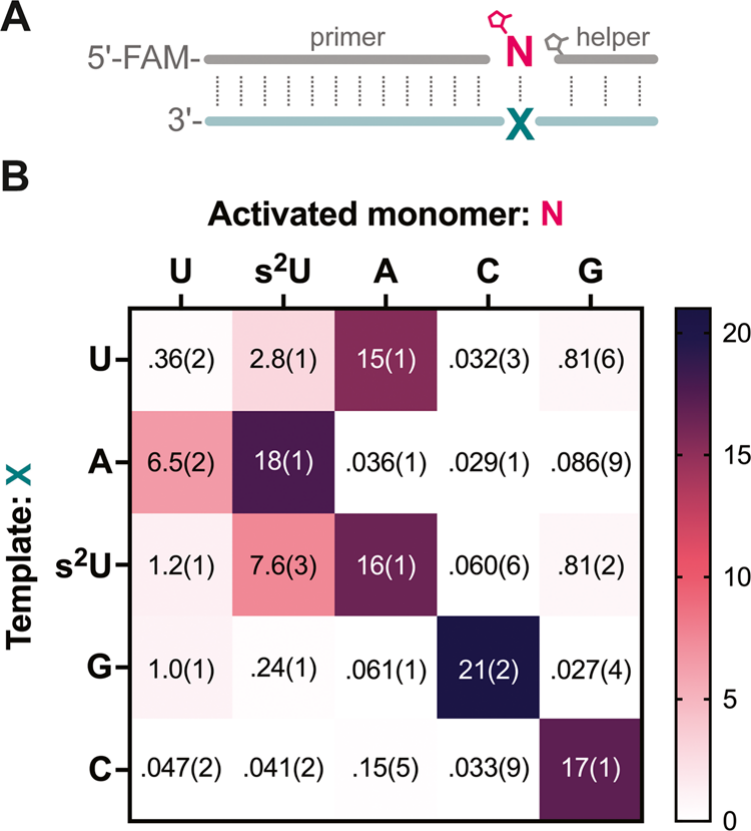
Nonenzymatic primer extension rates with base-pairing between s^2^U, U, A, C, and G. (A) Schematic representation of nonenzymatic primer extension with an activated mononucleotide (*N) and an activated trinucleotide helper. (B) Pseudo-first-order reaction rate constants (h^–1^) of primer extension with different *N across different templates: N = U, s^2^U, A, C, G and X = U, A, s^2^U, G, C. All reactions were performed at room temperature with 1.5 μM primer, 2.5 μM template, 20 mM *N, 0.5 mM activated helper, 100 mM MgCl_2_, and 200 mM Tris-HCl pH 8.0. Standard errors (N ≥ 3) are reported at the appropriate significant digit in parentheses.

Given our thermodynamic and crystallographic studies, which suggest that the reduced desolvation penalty and preorganization of the single-stranded RNA are likely the dominating factors for the stabilizing effect of s^2^U:s^2^U, we next asked whether the strong s^2^U:s^2^U base-pairing would affect the fidelity of nonenzymatic RNA copying. To do this, we prepared a complete set of 2-aminoimidazole (2AI or *) activated mononucleotides, and a series of primer extension templates that contain either the canonical nucleotides or s^2^U at the substrate binding site. The model system we used also contained 0.5 mM of a 2AI-activated downstream trinucleotide, which acts as a helper to catalyze the primer extension reaction (Figure 3A).^34,35^ The activated mononucleotide of interest can react with this activated trinucleotide to form a highly preorganized monomer-bridged trimer intermediate that greatly enhances primer extension reactions (Figure S5A). Without the facilitation of the activated trinucleotide helpers, some reactions with mismatched base pairs are barely detectable. Although bridged intermediate formation and the primer extension reactions in this model system are two separate sequential processes, the first step is relatively fast. As a result, we were able to measure pseudo-first-order reaction rate constants (h^–1^) as indicators of the efficiency of nonenzymatic primer extension.

To investigate the impact of the s^2^U:s^2^U base pair on the fidelity of nonenzymatic RNA copying, we evaluated the rate of nonenzymatic primer extension with the s^2^U:s^2^U mis-pair compared to all of the other possible base pairs (Figure 3A). In some of these reactions, we noticed a small percentage of mismatched primer–trimer ligation. The percentage of mismatched ligation is small in most cases and does not interfere with the measurement of the pseudo-first-order reaction rate constant for primer extension to the +1 product. However, when the template nucleotide adjacent to the primer is X = C, such that the first base of the activated trinucleotide *GAC can base pair with X = C, we observed higher levels of mismatched ligation products. The fact that both *N and *GAC are competing for the binding site X in this scenario interferes with the accurate measurement of the rate of mismatched primer extension. We avoided this problem by changing the downstream activated trinucleotide helper to *AGG and changing the template sequence accordingly when X = C. Since both *AGG and *GAC started with a purine and have two G:C base pairs, the overall binding affinity of the trinucleotide helper and the stacking interaction between the helper and the monomer should be similar. By changing the activated helper, we were able to obtain more accurate measurements of the rates of mismatched primer extension, with the caveat that the downstream template and helper sequences are different.

The results of our kinetic study show that when only primer extension with correct Watson–Crick base pairs is considered, substituting U with s^2^U can significantly reduce sequence biases, leading to remarkably similar rates of copying for s^2^U:A and C:G base pairs (Figure 3B). The enhanced rate and reduced sequence biases are the major benefits that originally led us to consider a role for this noncanonical nucleotide in prebiotic chemistry.^13,17^ However, the rapid copying of s^2^U in the template with an s^2^U substrate (Figure 3B) raised concerns about the fidelity of copying of s^2^U-containing templates.

We propose two possible explanations for the fast copying of an s^2^U template with an s^2^U substrate in this model system. One possibility is that the enhanced stability of the s^2^U:s^2^U base pair allows for better binding of the substrate to the primer-template complex. Alternatively, the fast mismatched primer extension could reflect the intrinsic reactivity of s^2^U, as thio-modification accelerates the incorporation of activated s^2^U not only across s^2^U but also across U and A templates (Figure 3). Previous kinetic studies on primer extension reactions have revealed that activated nucleotides with greater occupancy of the C3′-endo conformation exhibit a faster primer extension. This applies not only to activated s^2^U but also to other 2′-modified activated nucleotides.^17^ To investigate whether one or both of these mechanisms contribute to the s^2^U:s^2^U mismatched extension, we carried out further kinetic studies.

### Kinetic Parameters of Primer Extension with Presynthesized Bridged Dinucleotide Intermediates

To understand the reasons for the high rate of s^2^U:s^2^U mismatched nonenzymatic primer extension, we used a model system with presynthesized imidazolium-bridged intermediates (N*N) and defined template binding sites so that the measured kinetic parameters solely represent the +1 primer extension reaction (Figure S5B).^17^ We used bridged dinucleotides because they are highly reactive intermediates,^36,37^ and their synthesis and purification are much easier than for monomer-bridged-trimer intermediates with s^2^U modifications. The downstream blocking oligonucleotide in this system facilitates substrate binding but has a 5′–OH bond and cannot react with the substrate. This model allows us to evaluate the reactivities and binding affinities of different N*N substrates by measuring their primer extension rates at different concentrations and fitting the data to the Michaelis–Menten equation.^17^

**Figure 4:**
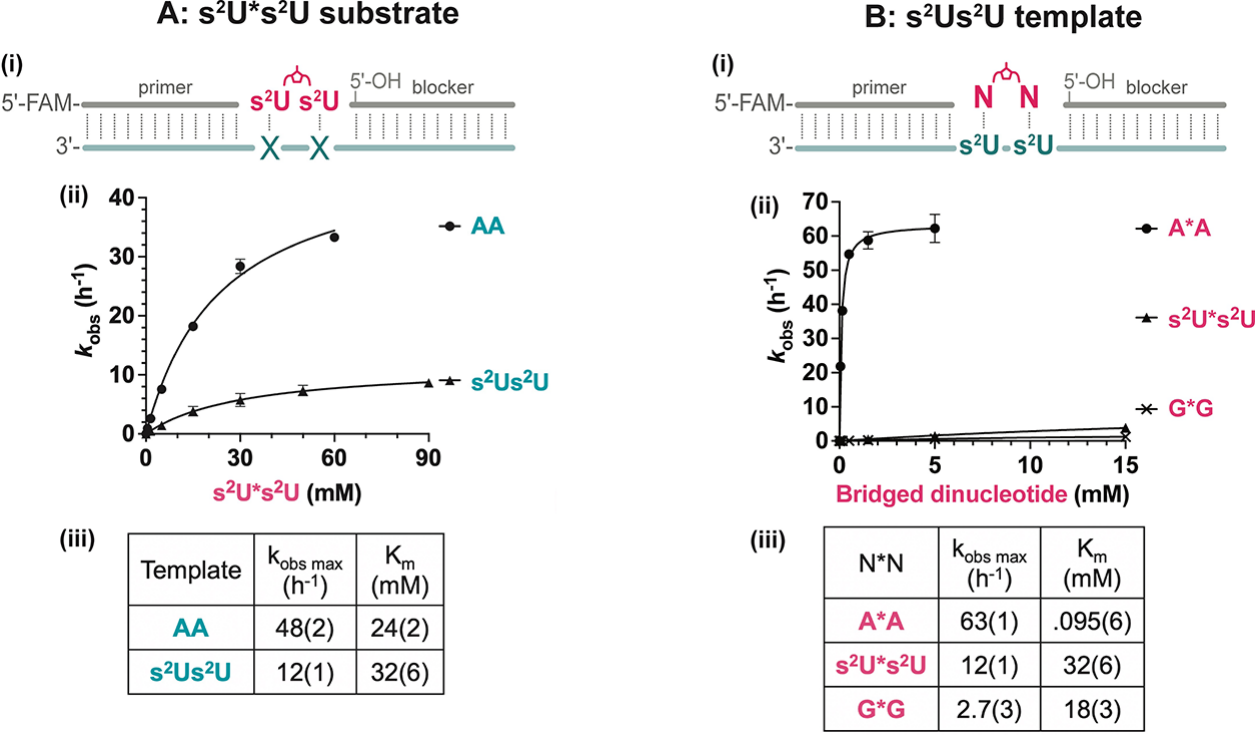
Kinetics of primer extension with s^2^U in either the substrate or the template. (A) Nonenzymatic primer extension with a 2-thiouridine imidazolium-bridged dinucleotide (s^2^U*s^2^U) in a primer-template-blocker complex. (i) Schematic representation. (ii) Michaelis–Menten curves for primer extension reactions with s^2^U*s^2^U across different templates. (iii) Kinetic parameters for extension with s^2^U*s^2^U on different templates. Part of the data was adapted from Figure S6 in ref (17) with permission under a Creative Commons Attribution 4.0 International License. Copyright 2022 Ding et al.; Published by Oxford University Press on behalf of Nucleic Acids Research. (B) Nonenzymatic primer extension with different bridged dinucleotides (N*Ns) on an s^2^Us^2^U template. (i) Schematic representation. (ii) Michaelis–Menten curves for primer extension reactions with different N*N substrates on the s^2^Us^2^U template. See also Figure S6. (iii) Kinetic parameters for extension with N*N on the s^2^Us^2^U template. All reactions were performed at room temperature with 1.5 μM primer, 2.5 μM template, 3.5 μM blocker, 100 mM MgCl_2_, and 200 mM Tris-HCl 8.0. Standard errors (N ≥ 3) are reported at the appropriate significant digit in parentheses.

We started by comparing primer extension with that of s^2^U*s^2^U over a template region consisting of either AA or s^2^Us^2^U (Figure 4Ai). The results suggested that primer extension on an s^2^Us^2^U template has about a 4-fold lower *k*_obs max_ and slightly higher *K*_M_ compared to the case with the AA template (Figure 4A). The slightly weakened binding is consistent with the reduced stacking interactions and weakened H-bond interactions, as seen in the crystal structure (Figures 1 and 2). We suggest that the diminished reactivity of the s^2^U*s^2^U substrate on an s^2^U*s^2^U template may be due to the non-Watson Crick geometry of the mismatched base pair, which could place the reactive phosphate in a position that is less favorable for primer extension.

To address the more prebiotically relevant scenario of competition between different substrates, we next evaluated the kinetics of nonenzymatic primer extension with different bridged dinucleotide substrates over an s^2^Us^2^U template (Figure 4Bi). Besides the Watson–Crick base paired A*A and mismatched s^2^U*s^2^U, we also measured primer extension with G*G because modest G extension over an s^2^U template was observed, as also observed in Figure 3. We find that primer extension with A*A greatly exceeds that of s^2^U*s^2^U and G*G, in terms of both reactivity and affinity (Figure 4B). The *k*_obs max_ for A*A is about 5-fold greater than that of s^2^U*s^2^U, but even more dramatically the *K*_m_ for A*A is approximately 300-fold lower than that of s^2^U*s^2^U, likely due to the stronger stacking of purines. While G*G binds to the template slightly more tightly than s^2^U*s^2^U, likely also due to the stronger stacking interactions of purines, its reactivity is very poor, probably because of the suboptimal configuration of the s^2^U:G wobble pairs. As a result, if all substrates were present at similar concentrations, correct extension by forming an A:s^2^U base pair would greatly outcompete s^2^U:s^2^U and G:s^2^U mismatched primer extension.

### Competition between s^2^U, A, C, and G Substrates to Copy an s^2^U-Containing Template

**Figure 5:**
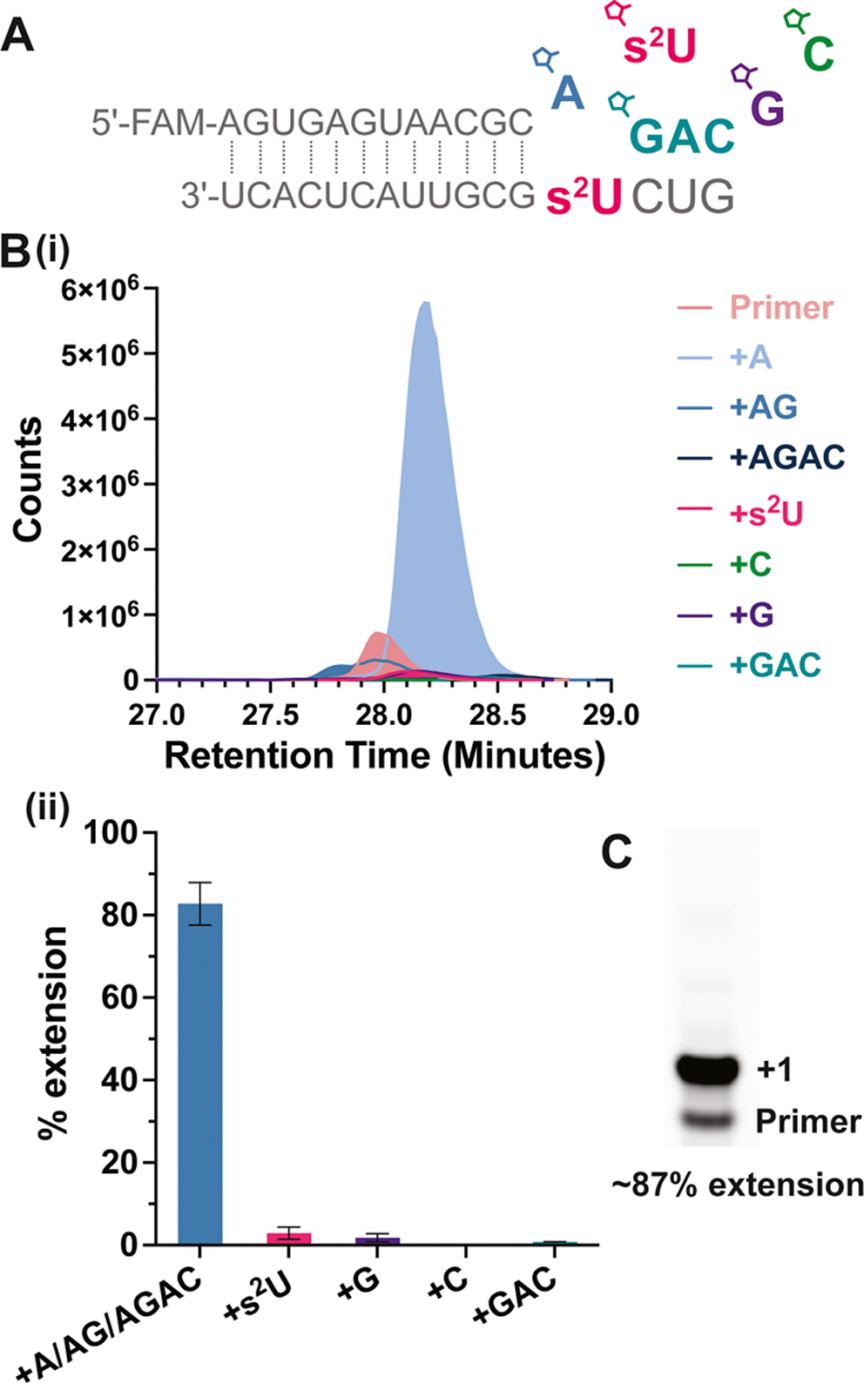
Competition between different activated mononucleotides on a template containing 2-thiouridine for nonenzymatic primer extension. (A) Schematic representation of the nonenzymatic primer extension with activated monomer mix (*s^2^U, *A, *C, and *G) and *GAC. (B) Quantification of the extension products through LC-MS. (i) A representative overlay of extracted ion chromatograms of residual FAM-primer and extension products observed by LC-MS from one of the trails. (ii) Quantitative analysis of the LC-MS. The + A, + AG, and + AGAC extensions are combined because all of them are the correct extension products through Watson–Crick base pairs. (C) PAGE gel analysis of the same extension reaction as in B(i). All +1 extensions overlap each other. All reactions were performed for 10 min with 20 mM total *N (5 mM *s^2^U, *A, *C, and *G), 0.5 mM *GAC, 100 mM MgCl_2_, and 200 mM Tris-HCl 8.0.

Given that all substrates will be competing for binding to all template sites during the copying of a mixed-sequence template, we sought to experimentally test whether s^2^U:s^2^U and G:s^2^U mis-pairs would induce significant errors during template copying. We focused our studies on the fidelity of copying a template s^2^U with a mixture of activated mononucleotides (*s^2^U, *A, *C, and *G) and an activated trinucleotide helper (*GAC) (Figure 5A). As described above, the activated mononucleotides first react with the activated trinucleotide, either spontaneously in solution or on the template,^38^ to form an N*GAC intermediate and then extend the primer by one nucleotide. Besides this mechanism, the primer may also extend by reaction with N*N substrates or by ligation of *GAC. The identity and extent of template copying products were determined by liquid chromatography–mass spectrometry (Figure 5B) and by PAGE gel electrophoresis, and we observed similar extension yields in both cases (Figure 5C).

The competitive primer extension experiments demonstrated relatively low levels of *s^2^U or *G extension over the s^2^U template. Similar levels of mismatched extensions were observed for s^2^U (3.3%) and G (2.0%). The misincorporation rate of 2AI-activated G on an s^2^U template is similar to that previously measured for 2-methylimidazole-activated s^2^U over a G template (1.6%).^13^ The overall error rate is better than what was observed with a 2AI-activated canonical mononucleotide mixture (8.5%) and similar to that with a canonical 2AI-bridged dinucleotide mixture (5.8%), reported in a sequencing study measuring the fidelity of primer extension on a random template at the 1 h time point.^39^ Our results imply that the low misincorporation rate of *s^2^U is because of its poor binding affinity to an s^2^U template, while the low incorporation rate of *G is due to both weak binding and low reactivity. As a result, primer extension with *A leads to the observed good fidelity. This is also consistent with our thermodynamic data showing that an s^2^U:s^2^U mis-pair is less stable than an s^2^U:A pair. Although this fidelity is not comparable with the fidelity of enzymatic DNA replication, it is approaching the fidelity needed for nonenzymatic RNA copying to transfer useful information.

### Stalling Effect of s^2^U:s^2^U at the 3′-End of the Primer

**Figure 6:**
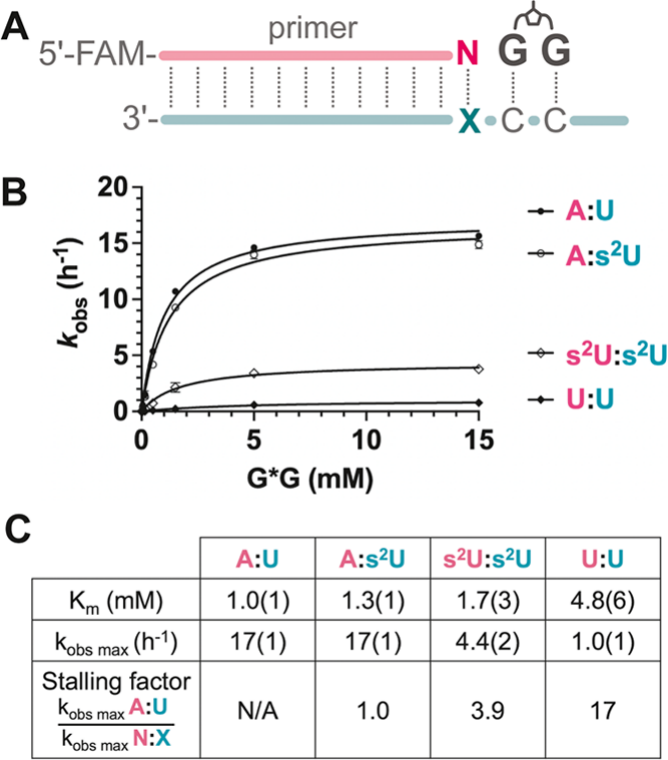
Stalling factors comparison between s^2^U and U. (A) Schematic representation of nonenzymatic primer extension of G*G with a primer that contained (N_1_) at the 3′ end, paired to *T*_1_ at the template. (B) Michaelis–Menten curves plotted for + G extension across different base pairs at the 3′ end of the primer. (C) Kinetic parameters for G*G from the Michaelis–Menten fitting and the stalling factors.

Previous studies have shown that a mismatch at the last primer-template base pair can significantly decrease the rate of subsequent primer extension.^40^ This stalling effect can significantly increase the effective fidelity of template copying. We therefore explored the influence of an s^2^U:s^2^U base pair at the 3′-end of the primer. For comparison, we also examined a primer ending with A, correctly paired with a template s^2^U, and the corresponding native forms (A:U and U:U) (Figure 6). We were pleased to find that primer extension after an s^2^U:s^2^U misincorporation significantly hinders the subsequent primer extension (stalling factor of ∼4 at saturating substrate). A U:U mismatch leads to an even more severe stalling factor of ∼17. In contrast, a terminal s^2^U:A primer/template base pair exhibits no stalling compared to a native A:U base pair. The significant stalling factor of s^2^U:s^2^U misincorporation would enhance the overall fidelity of RNA copying in the presence of s^2^U monomers.

## Discussion

The prebiotic chemistry of early Earth likely led to the synthesis of both the canonical nucleotides and certain modified nucleotides such as 2-thiouridine. Noncanonical nucleotides that have been preserved and still play roles in modern biology were likely selected based on their functionality, which may also have played a role during prebiotic chemistry. Our lab has previously described the higher rate and fidelity of nonenzymatic primer extension when using 2-thio-modified uridine as the substrate to copy A- and G-rich templates.^13^ However, the discovery that an s^2^U:s^2^U base pair can significantly stabilize RNA duplexes led us to revisit the fidelity of nonenzymatic copying involving s^2^U. Our kinetic analysis, competition experiments, and stalling effect measurements have confirmed that good fidelity can be maintained despite the stabilizing effect of s^2^U:s^2^U pairing within an RNA duplex.

The dominant mechanisms contributing to the greater stability of the s^2^U:s^2^U base pair compared to U:U likely act prior to the actual hybridization events, as our crystallographic studies indicate similar or slightly weaker H-bonding and stacking interactions with thio-modified U. We suggest that the stabilization derives in part from the conformational rigidity of s^2^U, which tends to preorganize single-stranded RNA in the A-form geometry that is characteristic of RNA duplexes.^9,29^ Such A-form stabilized RNA with its characteristic C3′-endo sugars is known to form a more stable duplex, as seen with LNA^41^ and 2′-OMe RNA.^42^ Additionally, the weaker H-bond ability of s^2^U may reduce the energetic penalty for the dissociation of bound water and, subsequently, reduce the cost of removing neighboring water molecules in the H-bonding network. The reduced desolvation penalty makes the hybridization event more enthalpically favored, thus stabilizing the duplex formation. Both the effect of rigidity and the weaker water interaction are capable of affecting bases adjacent to s^2^U, leading to significantly greater −Δ*G* values of hybridization.

The stabilizing effect of an s^2^U:s^2^U base pair within an RNA duplex is comparable to that of an A:U base pair; it is strongly stabilizing relative to a U:U mismatch, but much less stabilizing than an s^2^U:A Watson–Crick base pair. Our previous study demonstrated that the s^2^U:A base pair forms two strong Watson–Crick hydrogen bonds, exactly the same as the U:A pair.^31^ The hydrogen bonds in s^2^U:A do not involve the sulfur atom which is a weaker hydrogen bond acceptor, and the hydrogen bond lengths (2.8 and 2.9 Å) are both shorter than the sulfur-mediated hydrogen bond in s^2^U:s^2^U (Figures 1 and 2). In addition, due to the larger purine ring system, the s^2^U:A pair leads to stronger stacking interactions than the s^2^U:s^2^U pair.

In the context of primer extension, the substrate:template s^2^U:s^2^U base pair is not embedded in the middle of the RNA duplex and it has to compete with the stronger s^2^U:A base pair. During mismatched primer extension involving the s^2^U:s^2^U base pair, one s^2^U is an incoming mononucleotide substrate and the other is in the template adjacent to the annealed primer. As a result, neither the preorganized C3′-endo conformation nor the weaker bound water network of s^2^U in the single-stranded RNA can play a significant role in the case of primer extension. Previous studies have also indicated significantly less duplex stabilization from terminal s^2^U modifications compared to internal ones.^30^

In our study, we initially observed significant misincorporation of s^2^U over an s^2^U template when using a system composed of only the activated s^2^U monomer and a downstream activated trimer catalyst. The observed primer extension is likely due to the good intrinsic reactivity of a substrate with a C3′-endo conformation, the assistance of the downstream trinucleotide helper for better binding, and the saturation of the template with the reactive s^2^U bridged trinucleotide species. However, this experiment did not reflect the competitive nature of mixed-sequence template copying. Incorporation of the correct nucleotide A over an s^2^U template benefits from the high reactivity and strong binding due to the strong Watson–Crick base pair and strong stacking interactions. Primer extension over an s^2^U template with competition between *s^2^U, *A, *C, *G, and the *GAC helper resulted in a low level of s^2^U and G misincorporation, indicating good fidelity even in the presence of possible s^2^U:s^2^U mis-pair. Although some misincorporation of s^2^U does occur, the stalling effect will impede further extension of that mutated sequence.

Lastly, although we have focused on the application of the s^2^U modification to prebiotic RNA chemistry, the unique s^2^U:s^2^U base pair may have implications in modern biology and for drug design. Since an internal s^2^U:s^2^U base pair can be comparable with the U:A base pair, it may provide more options for the design of antisense compounds and RNA-based medications.^30,43,44^

## Conclusions

The 2-thiolation of uridine has been demonstrated to confer improved efficiency and fidelity for codon translation, ribozyme-catalyzed RNA copying, and nonenzymatic RNA copying. However, we discovered that s^2^U forms an unusually stable self-pair, that is almost as stabilizing as an A:U base pair within an RNA duplex. This unusual s^2^U:s^2^U interaction had not been considered in previous studies and raised concerns about the fidelity of template copying in the presence of activated s^2^U monomers. We therefore conducted thermodynamic and crystallographic studies to investigate the factors contributing to the stability of the s^2^U:s^2^U base pair. Our findings suggest that thiolation of the uridine bases likely reduces the desolvation penalty and preorganizes the single-stranded RNA, so as to favor hybridization. However, after hybridization, the duplex bearing the s^2^U:s^2^U base pair has similar or even weaker H-bonding and -stacking interactions at the modification sites. Our kinetic analyses of primer extension suggest that s^2^U is intrinsically reactive for nonenzymatic primer extension because of its C3′-endo conformation, but the s^2^U substrate binds only weakly to another s^2^U on the template next to the annealed primer. Finally, we used competition experiments to examine the effect of s^2^U:s^2^U on nonenzymatic RNA copying in the presence of all of the activated substrate nucleotides. We observed that an incoming A substrate strongly outcompetes an s^2^U substrate when copying an s^2^U template. Importantly, even if some s^2^U is misincorporated opposite s^2^U on the template, the strong stalling effect reduces the subsequent extension of the mutant strand. Therefore, we believe that s^2^U is still a promising candidate for enhancing nonenzymatic RNA copying with minimal cost in fidelity resulting from s^2^U:s^2^U mismatches.

## Supporting Information Available

Additional experimental details, materials, and methods for crystallization; crystal data collection, structure determination, and refinement; melting temperature measurements; synthesis of oligonucleotides and activated nucleotides; nonenzymatic primer extension reactions; primer extension competition experiments; duplex design and melting curves measurements; melting curves; linear least-squared fits of Van’t Hoff plots; overlap areas of all base steps in duplexes; base pair steps in duplexes; mechanism of nonenzymatic primer extension; Michaelis–Menten curves; crystallization condition; data collection statistics; structure refinement statistics; local base pair parameters for UU1; and local base pair step parameters for UU1 (PDF)

## Abbreviations

s^2^U: 2-thiouridine
PAGE: polyacrylamide gel electrophoresis
