# Supplementary material for: Unusual Base Pair between Two 2-Thiouridines and Its Implication for Nonenzymatic RNA Copying
Source: J Am Chem Soc. 2024 Jan 31;146(6):3861–71. doi: 10.1021/jacs.3c11158 (PMC10870715; doi:10.1021/jacs.3c11158)
Supplement: Supplementary file 1 — ja3c11158_si_001.pdf [file ja3c11158_si_001.pdf]

Supplementary Information

An Unusual Base Pair Between Two 2-Thiouridines and its Implication for Nonenzymatic RNA Copying

Dian Ding[1,2†], Ziyuan Fang[3†], Seohyun Chris Kim[1,2,4#], Derek K. O'Flaherty[5], Xiwen Jia[1,2], Talbot B. Stone[6,7], Lijun Zhou[6,7*], and Jack W. Szostak[3*]

[1]Department of Chemistry and Chemical Biology, Harvard University, 12 Oxford Street, Cambridge, Massachusetts 02138, USA.

[2]Department of Molecular Biology and Center for Computational and Integrative Biology, Massachusetts General Hospital, 185 Cambridge Street, Boston, Massachusetts 02114, USA.

[3]Howard Hughes Medical Institute, Department of Chemistry, The University of Chicago, Chicago, Illinois 60637, USA.

[4]Department of Genetics, Harvard Medical School, 77 Avenue Louis Pasteur, Boston, Massachusetts 02115, USA.

[5]Department of Chemistry, College of Engineering and Physical Sciences, University of Guelph, Guelph, Ontario N1G 2W1, Canada.

[6]Department of Biochemistry and Biophysics, Perelman School of Medicine, University of Pennsylvania, Philadelphia, PA 19104, USA.

[7]Penn Institute for RNA Innovation, University of Pennsylvania, Philadelphia, PA 19104, USA.

*To whom correspondence should be addressed; Email: jwszostak@uchicago.edu, lijun.zhou@pennmedicine.upenn.edu

†These authors contributed equally to this work.

Present address: [#] S.C.K.: Columbia University, New York, New York 10032, USA.

Materials and Methods

General information

All chemicals were purchased from Sigma-Aldrich (St. Louis, MO) and used without purification unless otherwise noted. 2-Thiouridine was purchased from Cayman Chemical (Ann Arbor, MI). 2,2′-Dipyridyl disulfide was purchased from Combi-Blocks (San Diego, CA). 1-Hydroxy-7-azabenzotriazole was purchased from Creosalus (Louisville, KY). Phosphoramidites and reagents used for solid-phase RNA synthesis were purchased from ChemGenes (Wilmington, MA) and Glen Research (Sterling, MA). Deuterated solvents were purchased from Cambridge Isotope Laboratories (Tewksbury, MA).

Reverse phase flash chromatography was performed using a prepacked RediSep Rf Gold C18Aq 50 g column from Teledyne Isco (Lincoln, NE). Preparatory-scale high performance liquid chromatography (HPLC) was carried out on an Agilent 1290 HPLC system, equipped with a preparative-scale Agilent ZORBAX Eclipse-XDB C18 column (21.2x250mm, 7 μm particle size). Purity of synthesized products was determined either by NMR or high-resolution mass spectrometry (HRMS). ^{1}H and ^{31}P spectra were acquired on a Varian Oxford AS-400 NMR spectrometer (400 MHz for ^{1}H, 162 MHz for ^{31}P) at 25 °C. HRMS was carried out on an Agilent 6520 QTOF LC-MS.

Crystallization

0.33 mM self-complementary RNA sequences in nuclease-free water (Invitrogen, Waltham, MA) were heated up to 90 °C for 2 min and then slowly cooled to room temperature. Crystal Screen HT, Index HT, Natrix HT (Hampton Research, Aliso Viejo, CA) and Nuc-Pro HTS (Jena Bioscience, Jena, Germany) were used to screen crystallization conditions at 20 ˚C using the sitting-drop vapor diffusion method. An NT8 robotic system and Rock Imager (Formulatrix, Waltham, MA) were used for crystallization screening and monitoring the crystallization process. Optimal crystallization conditions are listed in the Supplementary Table S1.

Crystal data collection, structure determination and refinement

Diffraction data were collected at a wavelength of about 1 Å (exact values are in Table S2) under a liquid nitrogen stream at 99 K on Beamline 201 at the Advanced Light Source in the Lawrence Berkeley National Laboratory (USA) or Beamline 23ID-B at the Advanced Photon Source in the Argonne National Laboratory (USA). The crystals were exposed for 0.2 s per image with a 0.2 Å oscillation angle. The distances between detector and the crystal were set to 180–300 mm. The data were processed by HKL2000[1] or xia2[2] and DIALS. The structures were solved by molecular replacement by PHASER[3] using the structure of 3ND4 as the search model[4]. All structures were refined by Refmac5 in CCP4i[5]. After several cycles of refinement, some water molecules and metal atoms were added in Coot[6]. Data collection, phasing, and refinement statistics of the determined structures are listed in Supplementary Table S2 and S3.

Synthesis of oligonucleotides

The oligonucleotides for thermodynamic studies were synthesized on K&A H-8-SE - Oligo Synthesizer, with phosphoramidites from ChemGenes (Wilmington, MA) and reagents from Glen Research (Sterling, MA). The oligonucleotides were cleaved from the solid support and deprotected with AMA at room temperature for two hours. The mixtures were lyophilized, then the 2′-TBDMS protecting group was removed by treatment with triethylamine trihydrofluoride (room temp. overnight for s^2U-containing oligonucleotides, 65°C 2.5 hours for canonical oligonucleotide) and purified by Glen-Pak cartridge column. The purity of oligonucleotides was confirmed by Agilent 6230 TOF LC-MS; further purification by analytical HPLC was used if needed.

The oligonucleotides used for crystallography and for nonenzymatic primer extension studies were prepared on an Expedite 8909 DNA/RNA synthesizer with phosphoramidites from ChemGenes (Wilmington, MA) and reagents from Glen Research (Sterling, MA). Deprotection and purification methods were as above, except that the canonical RNAs for crystal studies were purified by PAGE.

The canonical oligonucleotides used for nonenzymatic primer extension studies were purchased from Integrated DNA Technologies (Coralville, IA).

Melting temperatures

Melting temperatures were measured using an Agilent Cary 3500 UV-Vis Spectrophotometer. For each pair of complementary oligonucleotides, samples were prepared with the desired

concentration of the target oligonucleotide in 10 mM Tris-HCl (pH 8.0), 1 M NaCl and 2.5 mM EDTA. 200 μL mineral oil was added to the top of the RNA solution in the cuvette to prevent the evaporation of water. Melting curves were collected by following absorbance at 260 nm as a function of temperature using a temperature ramp of 0.5-1°C/min. The readings were collected in heating-cooling cycles with respect to a control sample containing 10 mM Tris-HCl (pH 8.0), 1 M NaCl and 2.5 mM EDTA. The melting temperatures were calculated from the interpolation of sigmoidal curves. Each experiment was repeated eight times, including four sets of data from low to high temperature and four sets of data from high to low temperature.

Synthesis of 2-aminoimidazolium activated mononucleotides and bridged dinucleotides

The synthesis was performed following previously published procedures.[7]

The activated mononucleotides ***A**, ***C**, ***U**, ***G**, and ***s^{2}U** were purified by reverse-phase flash chromatography, with a 50 g C18Aq column, over 12CVs of 0-10% acetonitrile in 2 mM TEAB buffer (pH 8.0). The fractions containing desired products were adjusted to pH 9.5-10 with NaOH, aliquoted into Eppendorf tubes, and lyophilized.

The bridged dinucleotides **A*A**, **G*G**, and **s^{2}U*s^{2}U** were purified by preparative scale HPLC using a C18 reverse phase column eluted over 20 mins in a gradient of 2-10% acetonitrile in 2 mM TEAB buffer (pH 8.0), under a flow rate of 15 mL/min. The fractions containing desired products were adjusted to pH 8 and with HCl, aliquoted into Eppendorf tubes, and lyophilized.

Synthesis of 2-aminoimidazole activated trimers *GAC & *AGG

The 5′-phosphorylated trimers were prepared by solid phase synthesis on a MerMade 6 DNA/RNA synthesizer, deprotected, and purified by reverse phase flash chromatography, with a 50 g C18Aq column, over 12 CVs of 0-10% acetonitrile in 2 mM TEAB buffer (pH 8.0). After lyophilizing to dryness, the 5′-phosphorylated trimers were added to 40 equivalents of 2AI and TPP, and dissolved in dry DMSO with 400 equiv. of TEA. The solution was then added to 40 equiv. of DPDS and incubated at room temperature for 6 hours. The product was then precipitated and purified with the same procedure as the activated mononucleotides.

Nonenzymatic primer extension reactions

The primer/template or the primer/template/blocker complexes were prepared in an annealing buffer with 5X final concentration: 7.5 μM primer, 12.5 μM template, 17.5 μM downstream

blocker if needed, 50 mM Tris-Cl pH 8.0, 50 mM NaCl, and 1 mM EDTA. The solution was heated at 85°C for 30 s and then slowly cooled to 25°C at a rate of 0.1°C/s in a thermal cycler machine. The annealed solution was then diluted into the primer extension reaction to concentrations of 1.5 μM primer, 2.5 μM template, 3.5 μM blocker (for the sandwich system), 200 mM Tris-Cl pH 8.0, and 100 mM $MgCl_2$.

For primer extension reactions with activated monomer and downstream activated trimer, the monomers were freshly prepared as a 50 mM stock solution and the activated trimer was freshly prepared as a 5 mM stock solution. In a 0.2 mL PCR tube, the primer/template reaction solution were place at the bottom, while the activated monomer and trimer stock solution were placed in the lid or on the wall, followed by immediate spin down to mix the solution and initiate the reaction. At each time point, 0.5 μL of reaction sample was added to 25 μL quench buffer containing 25 mM EDTA, 1X TBE, and 4 μM of an complementary RNA/DNA to the template in formamide. All reactions used the primer /FAM/AGU GAG UAA CGC. The template and complementary RNA sequence were listed as below (5′→ 3′).

Template: X	Activated trimer added	Template Sequence	Complementary RNA
U	*GAC	GUC U GCG UUA CUC ACU	AGU GAG UAA CGC A GAC
s^2U	*GAC	GUC s^2U GCG UUA CUC ACU	AGU GAG UAA CGC A GAC
A	*GAC	GUC A GCG UUA CUC ACU	AGU GAG UAA CGC U GAC
G	*GAC	GUC G GCG UUA CUC ACU	AGU GAG UAA CGC C GAC
C	*AGG	CCU C GCG UUA CUC ACU	AGU GAG UAA CGC G AGG

The Michalis-Menten experiment in Figure 4 was performed similarly, except that 2X stock solutions of bridged dinucleotides were freshly prepared and added to the primer/template buffer solution to initiate the primer extension reactions. All reactions used the primer 5′ - /FAM/AGU GAG UAA CGG and the blocker 5′-OH-G AUG UCA GAU AU. The template and complementary DNA sequence were listed as below (5′→ 3′).

Template: XX	Template Sequence	Complementary DNA
AA	AU AUC UGA CAU CAA CCG UUA CUC ACU	AGT GAG TAA CGG TTG ATG TCA GAT AT
s^2Us^2U	AU AUC UGA CAU C**s^2Us^2U** CCG UUA CUC ACU	AGT GAG TAA CGG AAG ATG TCA GAT AT

Primer extension competition experiments

The primer-template duplex was first annealed in a 16 μL solution containing 4 μM primer (/FAM/AGU GAG UAA CGC), 4.8 μM template (5′-OH-GUC s^2U GCG UUA CUC ACU), 50

mM Tris-HCl pH 8.0, 50 mM NaCl, and 1 mM EDTA. The solution was heated at 85°C for 30 s and then slowly cooled to 25°C at a rate of 0.1°C/s in a thermal cycler machine. The annealed product was then added to 16 µL of 1M Tris-HCl pH 8.0 and 8 µL 1 M $MgCl_2$. An equimolar mixture of *s^2U, *A, *C, and *G was freshly prepared at a 50 mM total concentration and the activated trimer *GAC was prepared at 5 mM. The reaction was initiated by adding the 32 µL of the *N stock and 8 µL of the *GAC stock to the primer-template solution to make the final reaction concentration of 5 µM primer, 6 µM template, 200 mM Tris-Cl pH 8.0, 100 mM $MgCl_2$, 20 mM *N (5 mM *s^2U, *A, *C & *G) and 0.5 mM *GAC.

After 10 min, the reaction mixture was added to 16 µL of 0.5 M EDTA, 16 µL of 5 M ammonium acetate, and 288 µL of cold ethanol. The solution was allowed to precipitate on dry ice for 1 h before being centrifuged at 15,000 rpm for 15 min, and then washed twice with 80% ethanol. The pellet was resuspended in LC-MS grade water and desalted by ion pairing reverse phase (IP-RP) purification on multiple C18 ZipTip pipette tips (Millipore, Billerica, MA). The tip was wetted with acetonitrile and then with 2M TEAA prior to sample binding. After extensive washing with 10 mM TEAA, the sample was eluted with 50% acetonitrile, dried under vacuum and resuspended in LC-MS grade water.

The eluted sample was loaded onto an Agilent 1200 HPLC coupled to an Agilent 6230 TOF equipped with a solvent degasser, column oven, autosampler, and diode array detector. The sample was separated by IP-RP-HPLC on a 100 mm × 1 mm Xbridge C18 column with 3.5 µm particle size (Water, Milford, MA) with (A) 200 mM 1,1,1,3,3,3-hexafluoro-2- propanol with 1.25 mM triethylamine, pH 7.0 and (B) methanol. The sample was eluted between 2.5% and 20% B over 28.5 min with a flow rate of 125 µL/min at 60°C. The sample was analyzed in negative mode from 239 m/z to 3200 m/z with a scan rate of 1 spectrum/s, drying gas flow of 8 L/min at 325°C, nebulizer pressure of 30 psig, capillary voltage of 3500V, fragmentor at 200 V, and skimmer at 65 V. Extracted ion chromatograms were generated with the Find by Formula algorithm in Agilent's MassHunter Qualitative Analysis software using a chemical formula database of possible primer extension products. The database was generated by calculating the composition of all possible random RNA extension products.

References

(1) Otwinowski, Z.; Minor, W. Processing of X-Ray Diffraction Data Collected in Oscillation Mode. *Methods Enzymol* **1997**, *276*, 307–326.

(2) Winter, G. Xia2: An Expert System for Macromolecular Crystallography Data Reduction. *J Appl Crystallogr* **2010**, *43* (1), 186–190.

(3) McCoy, A. J.; Grosse-Kunstleve, R. W.; Adams, P. D.; Winn, M. D.; Storoni, L. C.; Read, R. J. Phaser Crystallographic Software. *J Appl Crystallogr* **2007**, *40* (4), 658–674.

(4) Mooers, B. H. M.; Singh, A. The Crystal Structure of an Oligo(U):Pre-MRNA Duplex from a Trypanosome RNA Editing Substrate. *RNA* **2011**, *17* (10), 1870–1883.

(5) Murshudov, G. N.; Vagin, A. A.; Dodson, E. J. Refinement of Macromolecular Structures by the Maximum-Likelihood Method. *Acta Crystallogr D Biol Crystallogr* **1997**, *53* (3), 240–255.

(6) Emsley, P.; Lohkamp, B.; Scott, W. G.; Cowtan, K. Features and Development of Coot. *Acta Crystallogr D Biol Crystallogr* **2010**, *66* (4), 486–501.

(7) Ding, D.; Zhou, L.; Giurgiu, C.; Szostak, J. W. Kinetic Explanations for the Sequence Biases Observed in the Nonenzymatic Copying of RNA Templates. *Nucleic Acids Res* **2022**, *50* (1), 35–45.

Supplementary Discussion

We also conducted melting curves measurement for UU1, SS1, UU2 and SS2. We observed significant increase in the T_m with thiolation modification as expected. However, these RNAs form self-hairpin secondary structures in solution, so the melting data does not reflect just the melting temperature of the duplexes.

To avoid problems resulting from self-structures, we designed the sequences of the 9-bp RNA duplex in Table 1 with the assistance of NUPACK software. All duplexes contain the base-pair of interest in the middle, flanked by four Watson-Crick base-pairs. The NUPACK data shows that these sequences cannot form self-hairpin or homodimer structures.

Supplementary Figures and Tables

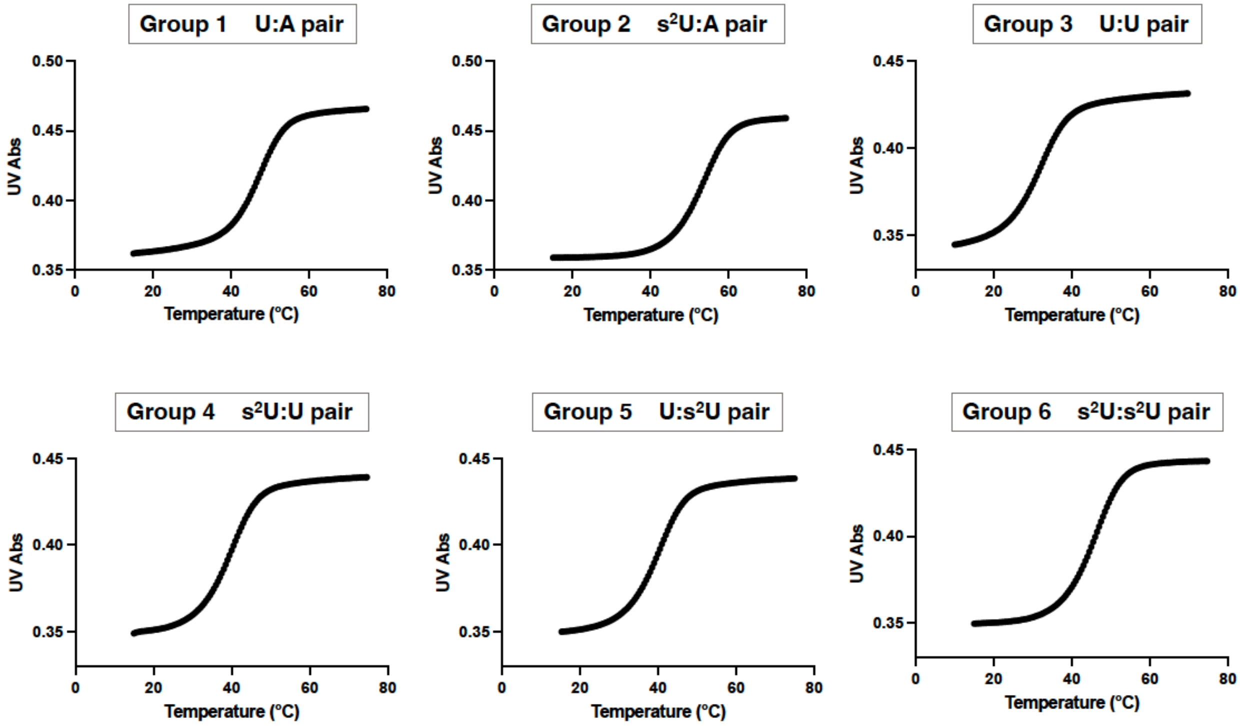

Figure S1. Representative melting curves collected during the thermal denaturation of 5 μM oligonucleotide in 10 mM Tris-HCl 8.0, 1 M NaCl, and 2.5 mM EDTA. See Table 1 of the main text for the sequences of duplexes.

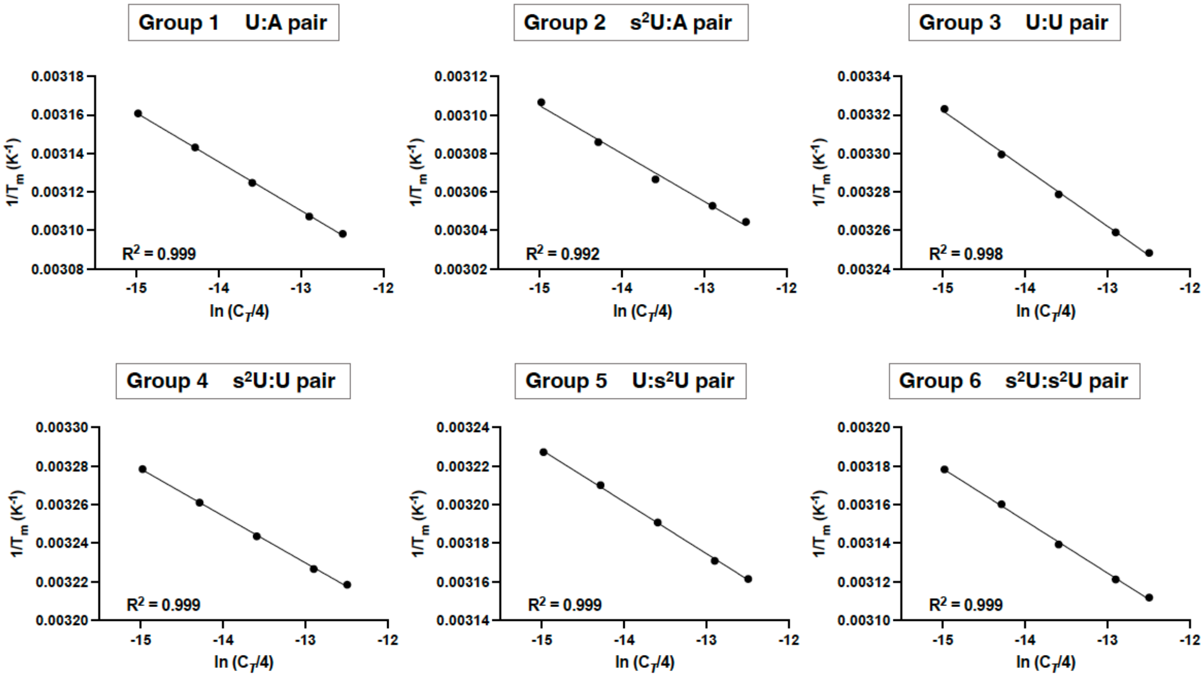

Figure S2. Linear least-squared fits of a Van't Hoff plot of inverse melting temperature (T_m^{-1}) collected from optical melts at different oligonucleotide concentrations. See Table 1 of the main text for the sequences of duplexes.

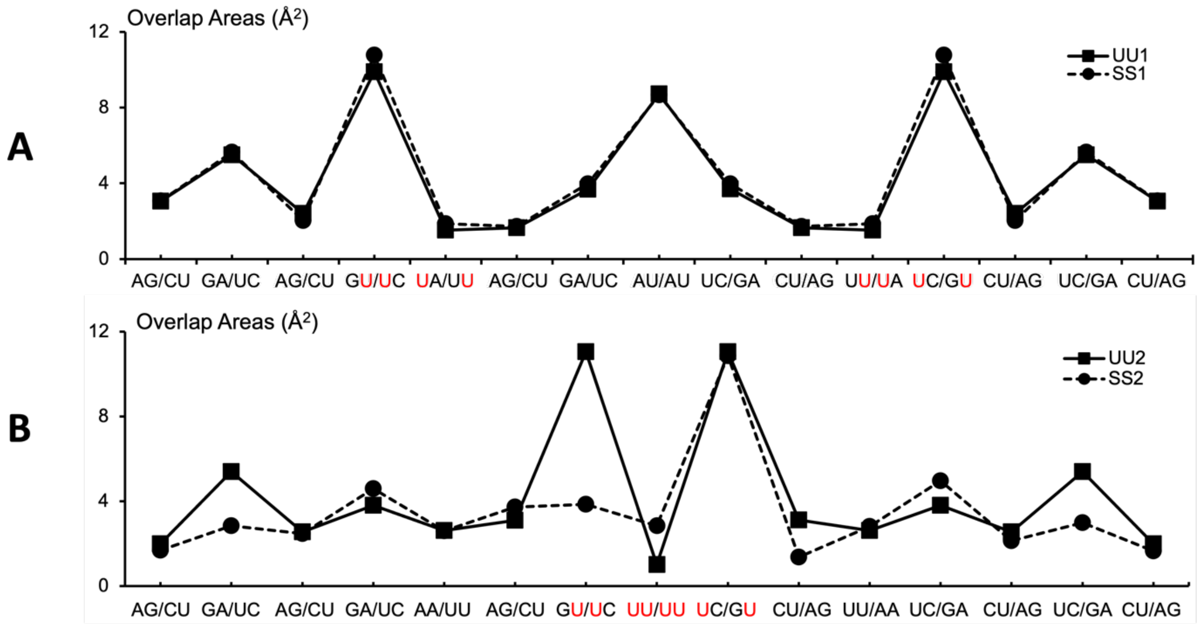

Figure S3. Overlap areas of all base steps in duplexes (A) UU1 and SS1 or (B) UU2 and SS2 (with s^2U modified on the red position).

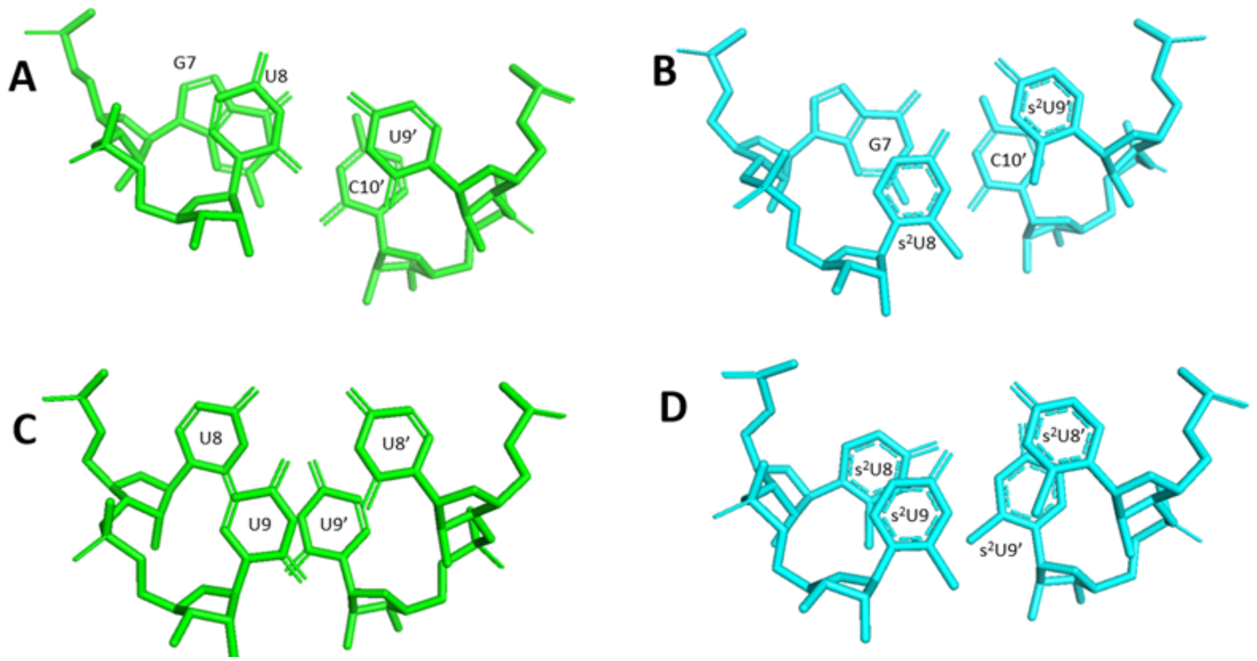

Figure S4. Base-pair steps close to the U:U pairs or s²U:s²U pairs in UU2 and SS2. (A) G7-U8/U9′ -C10′ step in UU2. (B) G7-U8/U9′ -C10′ step in SS2. (C) U8-U9/U8′ -U9′ step in UU2. (D) U8-U9/U8′ -U9′ step in SS2.

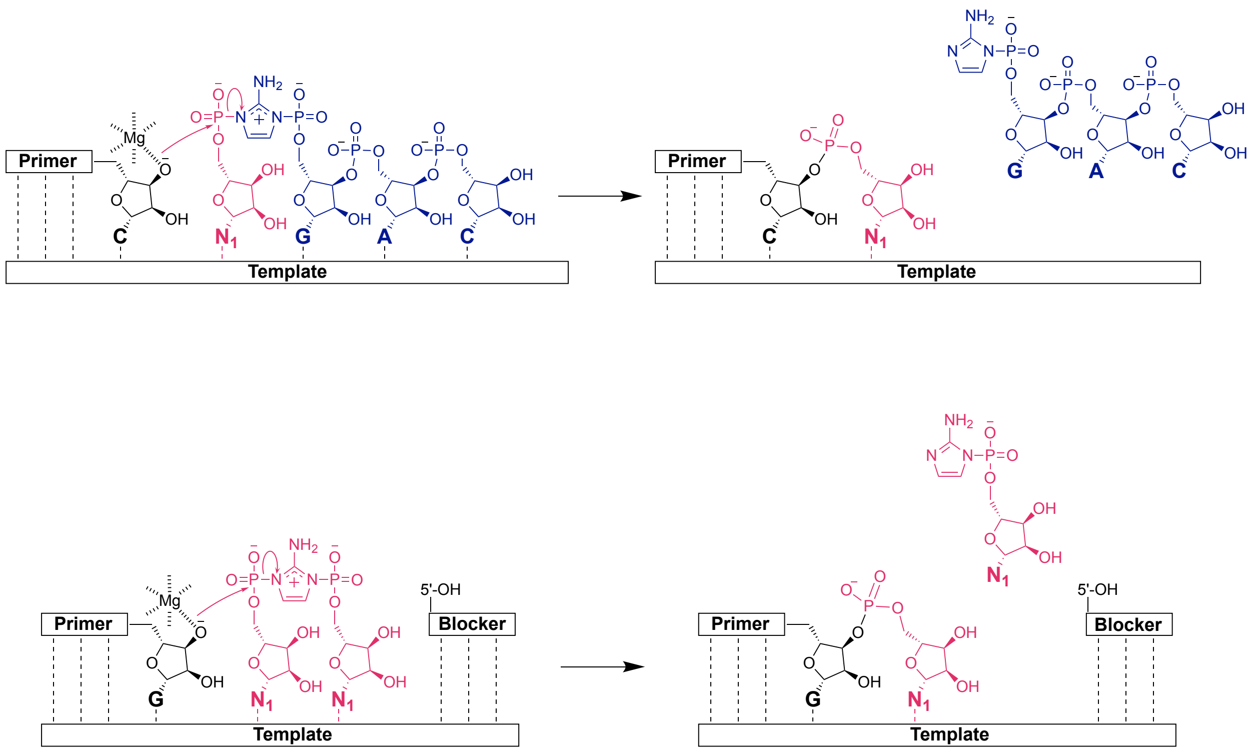

Figure S5. Mechanism of nonenzymatic primer extension reactions studied in this paper. (A) Primer extension through a monomer-bridged-oligonucleotide intermediate. (B) Primer extension through a bridged dinucleotide intermediate inside a primer-template-blocker system.

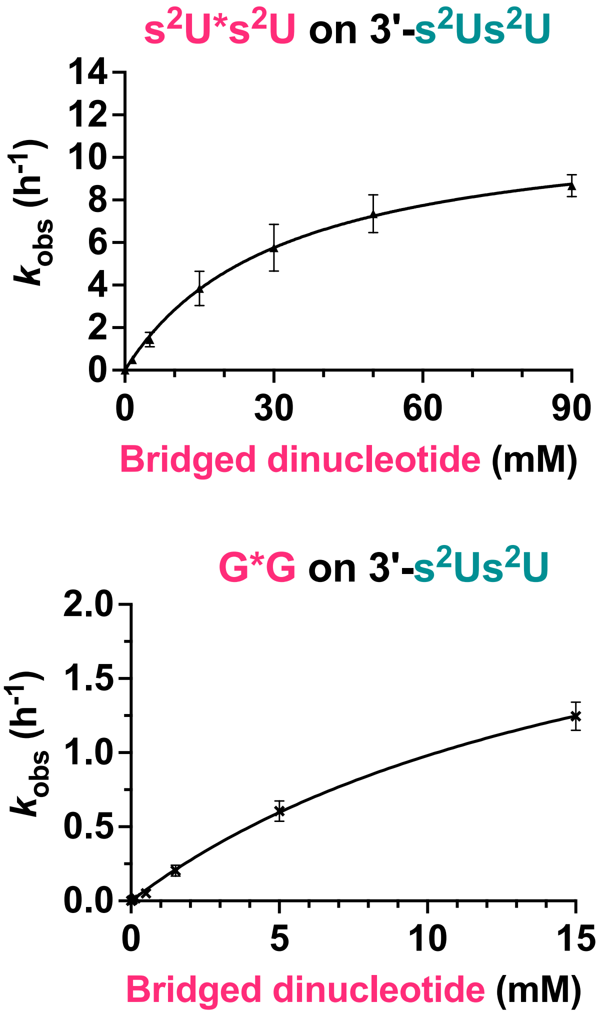

Figure S6. Michaelis-Menten curves plotted for s^2U*s^2U (top) and G*G (bottom) across a template containing 2-thiouiridine.

Table S1. Optimized Conditions for Crystallization

Sequence	Optimized crystallization conditions
UU1	80 mM Strontium chloride hexahydrate, 20 mM Magnesium chloride hexahydrate, 40 mM Sodium cacodylate trihydrate pH 7.0, 20% v/v (+/-)-2-Methyl-2,4-pentanediol, 12 mM Spermine tetrahydrochloride
SS1	2 M Lithium sulfate, 3 % w/v 2-Methyl-2,4-pentanediol
UU2	75 mM Sodium chloride, 2 mM Calcium chloride dihydrate, 50 mM Sodium cacodylate trihydrate pH 6.0, 30% w/v 1,6 Hexanediol, 5 mM Spermine
SS2	20 mM Magnesium chloride hexahydrate, 2 mM Cobalt (II) chloride hexahydrate, 50 mM HEPES sodium pH 7.5, 2.0 M Ammonium sulfate, 1 mM Spermine

Table S2. Data Collection Statistics

Sequence	UU1	SS1	UU2	SS2
PDB code	8FEO	8FEP	8FEQ	8FER
Beamline	23ID-B	23ID-B	2.0.1	2.0.1
Wavelength (Å)	1.033175	1.033175	1.037690	1.037690
Space group	R32	R32	R32	R32
Unit cell parameters (Å, °)	41.50, 41.50, 125.30, 90, 90, 120	41.72, 41.72, 126.23, 90, 90, 120	41.26, 41.26, 124.24, 90, 90, 120	43.81, 43.81, 258.17, 90, 90, 120
Resolution range (Å)	17.79-1.52 (1.55-1.52)	21.04-1.55 (1.58-1.55)	30.97-1.50 (1.53-1.50)	50-1.54 (1.57-1.54)
Unique reflections	6587 (322)	6354 (310)	6853 (352)	14744 (747)
Completeness (%)	98.3 (99.1)	98.5 (100)	100 (100)	99.7 (100)
R_{merge} (%)	8.1 (25.3)	6.6 (18.7)	2.9 (8.1)	11.3 (42.9)
$\langle I/\sigma(I) \rangle$	36.4 (3.0)	30.0 (2.1)	14.2 (2.3)	13.7 (4.4)

Table S3. Structure refinement statistics.

Sequence	UU1	SS1	UU2	SS2
PDB code	8FEO	8FEP	8FEQ	8FER
RNA strands per asymmetric unit	1	1	1	2
Resolution range (Å)	17.80-1.52	21.05-1.55	30.99-1.50	43.07-1.54
Number of reflections	6267	6026	6520	13496
R_{work} (%)	19.8	20.6	17.7	21.9
R_{free} (%)	23.9	26.9	21.0	25.9
Bond length R.M.S. (Å)	0.015	0.011	0.021	0.017
Bond angle R.M.S. (°)	2.56	2.55	2.58	2.76
Average B-factors (Å^2)	21.39	25.46	18.2	14.7

Table S4. Local base pair parameters for UU1.

Pair	Shear (Å)	Stretch (Å)	Stagger (°)	Buckle (°)	Propeller (°)	Opening (°)
A-U	-0.02	-0.11	0.06	0.12	-9.62	1.70
G-C	-0.18	-0.20	-0.17	-5.06	-15.12	-0.99
A-U	0.10	-0.08	0.01	-3.04	-14.30	2.07
G-C	-0.32	-0.18	-0.11	-5.11	-14.54	-0.16
U-U	2.33	-1.53	0.01	-2.43	-9.43	0.94
A-U	-0.15	-0.02	0.04	-5.44	-14.03	9.50
G-C	-0.14	-0.11	0.04	-2.40	-6.28	-1.15
A-U	-0.10	-0.05	-0.13	-0.78	-12.96	1.56
U-A	0.10	-0.05	-0.13	0.78	-12.96	1.55
C-G	0.15	-0.11	0.04	2.40	-6.28	-1.16
U-A	0.15	-0.02	0.04	5.44	-14.03	9.51
U-U	-2.33	-1.53	0.01	2.43	-9.44	0.94
C-G	0.32	-0.18	-0.11	5.11	-14.54	-0.17
U-A	-0.10	-0.08	0.01	3.04	-14.30	2.07
C-G	0.18	-0.20	-0.17	5.06	-15.12	-1.00
U-A	0.02	-0.11	0.06	-0.12	-9.62	1.69

Table S5. Local base pair step parameters for UU1.

Step	Shift (Å)	Slide (Å)	Rise (Å)	Tilt (°)	Roll (°)	Twist (°)	Overlap Area (Å^2)
AG/CU	-0.60	-1.10	3.34	-0.02	8.05	33.76	3.05
GA/UC	0.71	-1.15	3.17	0.41	4.69	33.74	5.50
AG/CU	-0.25	-1.41	3.28	-0.58	10.71	30.58	2.41
GU/UC	0.36	-1.19	3.32	0.21	7.79	41.81	9.89
UA/UU	1.12	-2.16	3.10	6.89	19.15	22.31	1.53
AG/CU	-0.83	-2.01	3.10	-0.99	6.32	25.92	1.65
GA/UC	-0.27	-1.51	3.24	1.07	7.39	31.86	3.69
AU/AU	0.00	-1.18	3.23	0.00	6.45	32.50	9.73
UC/GA	0.27	-1.51	3.24	-1.06	7.39	31.87	3.70
CU/AG	0.83	-2.01	3.10	0.99	6.32	25.92	1.65
UU/UA	-1.12	-2.16	3.10	-6.89	19.15	22.31	1.53
UC/GU	-0.36	-1.19	3.32	-0.21	7.79	41.81	9.89
CU/AG	0.25	-1.41	3.28	0.59	10.72	30.58	2.41
UC/GA	-0.71	-1.15	3.17	-0.41	4.69	33.75	5.50
CU/AG	0.60	-1.10	3.34	0.02	8.05	33.75	3.05

Table S6. Local base pair parameters for SS1.

Pair	Shear (Å)	Stretch (Å)	Stagger (°)	Buckle (°)	Propeller (°)	Opening (°)
A-U	0.00	-0.13	0.03	-1.31	-10.26	2.05
G-C	-0.20	-0.20	-0.11	-4.11	-14.54	-1.05
A-U	0.10	-0.15	0.06	-1.90	-15.87	0.57
G-C	-0.29	-0.22	-0.16	-6.36	-12.51	-1.46
U-U	2.29	-1.54	0.10	-1.77	-8.73	-1.54
A-U	-0.21	-0.05	-0.02	-5.35	-14.34	8.22
G-C	-0.18	-0.15	0.03	-2.41	-6.66	-1.34
A-U	-0.03	-0.15	-0.09	-1.51	-13.95	0.73
U-A	0.03	-0.15	-0.09	1.51	-13.95	0.73
C-G	0.18	-0.15	0.03	2.41	-6.66	-1.34
U-A	0.21	-0.05	-0.02	5.35	-14.34	8.22
U-U	-2.29	-1.54	0.10	1.77	-8.73	-1.55
C-G	0.29	-0.22	-0.16	6.36	-12.52	-1.47
U-A	-0.10	-0.15	0.06	1.90	-15.88	0.56
C-G	0.20	-0.20	-0.11	4.11	-14.54	-1.04
U-A	0.00	-0.13	0.03	1.30	-10.26	2.06

Table S7. Local base pair step parameters for SS1.

Step	Shift (Å)	Slide (Å)	Rise (Å)	Tilt (°)	Roll (°)	Twist (°)	Overlap Area (Å2)
AG/CU	-0.62	-1.09	3.24	-0.83	7.67	33.31	3.08
GA/UC	0.70	-1.15	3.16	0.66	4.80	33.67	5.64
AG/CU	-0.40	-1.42	3.33	-0.87	11.33	31.98	2.03
GU/UC	0.26	-1.20	3.22	-1.06	7.10	41.22	10.78
UA/UU	1.09	-2.32	3.06	7.73	18.68	22.06	1.87
AG/CU	-0.87	-1.93	3.12	-1.40	5.74	26.32	1.72
GA/UC	-0.20	-1.52	3.22	0.73	7.77	31.84	3.98
AU/AU	0.00	-1.19	3.16	0.00	5.76	32.09	8.67
UC/GA	0.20	-1.52	3.22	-0.73	7.77	31.84	3.97
CU/AG	0.87	-1.93	3.12	1.40	5.74	26.32	1.72
UU/UA	-1.09	-2.32	3.06	-7.72	18.68	22.05	1.87
UC/GU	-0.26	-1.20	3.22	1.06	7.10	41.22	10.78
CU/AG	0.40	-1.42	3.33	0.87	11.33	31.98	2.03
UC/GA	-0.70	-1.15	3.16	-0.66	4.80	33.67	5.64
CU/AG	0.62	-1.09	3.24	0.82	7.67	33.30	3.08

Table S8. Local base pair parameters for UU2.

Pair	Shear (Å)	Stretch (Å)	Stagger (°)	Buckle (°)	Propeller (°)	Opening (°)
A-U	0.07	-0.11	0.11	1.24	-7.94	0.60
G-C	-0.35	-0.25	-0.15	-3.81	-13.62	-1.59
A-U	0.01	-0.11	0.04	-0.87	-12.10	1.72
G-C	-0.17	-0.19	-0.22	-5.19	-13.83	-1.84
A-U	0.07	-0.09	-0.03	-7.04	-10.39	2.43
A-U	0.08	-0.08	-0.02	-2.55	-11.20	0.88
G-C	-0.15	-0.12	0.11	1.42	-13.57	1.64
U-U	2.39	-1.84	0.20	4.70	-17.49	12.83
U-U	-2.39	-1.84	0.20	-4.70	-17.50	12.83
C-G	0.15	-0.12	0.11	-1.42	-13.57	1.64
U-A	-0.08	-0.08	-0.02	2.54	-11.20	0.88
U-A	-0.07	-0.09	-0.03	7.04	-10.39	2.44
C-G	0.17	-0.19	-0.22	5.19	-13.83	-1.84
U-A	-0.01	-0.11	0.04	0.87	-12.09	1.73
C-G	0.35	-0.25	-0.15	3.81	-13.62	-1.59
U-A	-0.07	-0.11	0.11	-1.24	-7.93	0.60

Table S9. Local base pair step parameters for UU2.

Step	Shift (Å)	Slide (Å)	Rise (Å)	Tilt (°)	Roll (°)	Twist (°)	Overlap Area (Å2)
AG/CU	-0.60	-1.30	3.33	-0.17	6.22	34.12	1.99
GA/UC	0.52	-1.19	3.16	-0.66	5.47	33.71	5.39
AG/CU	-0.05	-1.64	3.39	0.52	10.42	30.32	2.56
GA/UC	0.32	-1.72	3.28	-1.34	11.30	31.93	3.80
AA/UU	0.23	-1.72	3.17	2.98	9.00	30.79	2.62
AG/CU	0.31	-2.27	3.09	0.13	9.28	22.64	3.10
GU/UC	0.57	-0.85	3.24	0.08	3.38	42.98	11.04
UU/UU	0.00	-2.17	2.96	0.00	12.52	20.63	1.02
UC/GU	-0.57	-0.85	3.24	-0.08	3.39	42.98	11.04
CU/AG	-0.31	-2.27	3.09	-0.13	9.28	22.64	3.11
UU/AA	-0.23	-1.72	3.17	-2.98	9.00	30.80	2.62
UC/GA	-0.32	-1.72	3.28	1.34	11.30	31.93	3.80
CU/AG	0.05	-1.64	3.39	-0.52	10.42	30.33	2.56
UC/GA	-0.52	-1.19	3.16	0.66	5.46	33.70	5.39
CU/AG	0.60	-1.30	3.33	0.18	6.22	34.13	1.99

Table S10. Local base pair parameters for SS2.

Pair	Shear (Å)	Stretch (Å)	Stagger (°)	Buckle (°)	Propeller (°)	Opening (°)
A-U	0.12	-0.12	-0.09	-1.44	-7.12	-5.52
G-C	-0.20	-0.18	-0.05	-0.69	-10.44	-2.59
A-U	0.12	-0.10	-0.11	-6.27	-8.44	0.74
G-C	-0.43	-0.20	-0.23	-9.37	-14.52	-1.11
A-U	0.06	-0.09	-0.11	-7.13	-8.23	1.69
A-U	0.08	-0.09	-0.04	1.45	-7.30	-2.96
G-C	-0.13	-0.23	0.08	0.38	-13.96	-1.42
U-U	-2.28	-1.66	-0.34	2.14	-13.44	-4.81
U-U	-2.21	-1.52	0.00	-2.68	-5.09	-1.48
C-G	0.30	-0.22	-0.14	1.35	-9.31	-1.69
U-A	0.06	-0.08	-0.04	3.58	-9.31	4.26
U-A	-0.08	-0.11	-0.12	4.32	-7.73	0.26
C-G	0.45	-0.23	-0.15	8.71	-12.42	-1.66
U-A	0.05	-0.11	-0.14	6.56	-6.12	3.95
C-G	0.31	-0.18	-0.28	4.93	-8.13	-2.09
U-A	0.04	-0.09	-0.39	12.44	1.82	3.46

Table S11. Local base pair step parameters for SS2.

Step	Shift (Å)	Slide (Å)	Rise (Å)	Tilt (°)	Roll (°)	Twist (°)	Overlap Area (Å2)
AG/CU	-0.33	-1.79	3.23	-2.82	8.24	29.84	1.69
GA/UC	-0.10	-1.89	3.37	-0.21	10.37	34.91	2.85
AG/CU	0.25	-1.87	3.34	3.08	12.42	27.78	2.48
GA/UC	0.46	-1.51	3.21	1.14	9.47	32.26	4.59
AA/UU	-0.31	-1.37	3.04	1.61	5.69	32.57	2.60
AG/CU	0.64	-1.86	3.20	0.21	9.01	26.30	3.73
GU/UC	-0.57	-1.85	3.00	-1.48	8.10	25.67	3.86
UU/UU	0.41	-1.40	3.52	-8.24	16.17	37.00	2.85
UC/GU	0.04	-1.33	3.22	1.11	7.72	39.85	10.85
CU/AG	0.93	-1.84	3.15	2.99	7.96	28.57	1.36
UU/AA	-0.62	-1.86	3.24	-1.99	6.71	29.95	2.81
UC/GA	-0.57	-1.50	3.17	-2.66	4.76	31.85	4.96
CU/AG	0.14	-1.90	3.34	-2.21	8.24	29.83	2.14
UC/GA	-0.09	-1.93	3.31	1.43	10.43	33.04	2.99
CU/AG	0.59	-2.07	3.23	0.24	2.71	24.70	1.66
